# Supplementary material for: Habitat use of Bechstein´s bats (Myotis bechsteinii) within wind parks in forests
Source: PLoS One. 2026 Mar 25;21(3):e0344730. doi: 10.1371/journal.pone.0344730 (PMC13016307; doi:10.1371/journal.pone.0344730)
Supplement: S1 File — Table S2 Supporting information on the roosts of the Bechstein´s bats: ID, species, condition, roost height, distance to next turbine, x and y coordinates Table S3 S3a -S3d: Roost use of the tagged animals and results of emergence counts. Table S4: Size of individual MCPs and number and size of 95%-LoCoH and 50%-LoCoH 50% for all tagged animals. (DOCX) [file pone.0344730.s001.docx]

**Table S1:** Overview over the tagged Bechstein´s bats

| **Animal ID** | **Study area** | **Reproductive state** | **Tracking period** | **Number of nights with cross bearings** | **Number of bearings** | **Number of valid positions** |
| --- | --- | --- | --- | --- | --- | --- |
| SH1 | Hunsrück | lactating | 30.6.-9.7.2019 | 2 | 155 | 132 |
| SH2 | Hunsrück | lactating | 3.7.-10.7.2019 | 2 | 96 | 76 |
| SH3 | Hunsrück | lactating | 3.7.-13.7.2019 | 2 | 135 | 104 |
| SH4 | Hunsrück | lactating | 6.7.-19.7.2019 | 2 | 143 | 111 |
| SH5 | Hunsrück | lactating | 8.7.-20.7.2019 | 3 | 160 | 123 |
| SH6 | Hunsrück | lactating | 11.7.-20.7.2019 | 2 | 112 | 89 |
| SH7 | Hunsrück | lactating | 16.7.-20.7.2019 | 2 | 128 | 110 |
| SH8 | Hunsrück | lactating | 16.7.-20.7.2019 | 2 | 190 | 168 |
| SH9 | Hunsrück | lactating | 29.06.-2.7.2022 | 2 | 169 | 131 |
| SH10 | Hunsrück | lactating | 29.6-2.7.2022 | 2 | 104 | 68 |
| SH11 | Hunsrück | lactating | 5.7.-15.7.2022 | 3 | 130 | 110 |
| SH12 | Hunsrück | lactating | 5.7.-11.7.2022 | 3 | 131 | 96 |
| SH13 | Hunsrück | lactating | 10.7.-17.7.2022 | 2 | 115 | 78 |
| SH14 | Hunsrück | lactating | 12.7.-17.7.2022 | 2 | 139 | 110 |
| SH15 | Hunsrück | lactating | 15.7.-17.7.2022 | 3 | 121 | 94 |
| SP1 | Palatinate | lactating | 30.6.-4.7.2020 | 3 | 147 | 96 |
| SP2 | Palatinate | lactating | 3.7.-10.7.2020 | 3 | 179 | 128 |
| SP3 | Palatinate | Not reproductive | 9.7.-24.7.2020 | 2 | 176 | 135 |
| SP4 | Palatinate | lactating | 14.7.-24.7.2020 | 2 | 114 | 81 |
| SP5 | Palatinate | Not reproductive | 16.7.-29.7.2020 | 3 | 165 | 127 |
| SP6 | Palatinate | lactating | 16.7.-22.7.2020 | 4 | 168 | 146 |
| SP7 | Palatinate | lactating | 20.7.-29.7.2020 | 3 | 180 | 151 |
| SP8 | Palatinate | Not reproductive | 20.7.-29.7.2020 | 2 | 214 | 147 |
| SP9 | Palatinate | lactating | 26.6.-6.7.2023 | 4 | 159 | 100 |
| SP10 | Palatinate | lactating | 26.6.-5.7.2023 | 3 | 126 | 97 |
| SP11 | Palatinate | lactating | 28.6.-9.7.2023 | 3 | 166 | 99 |
| SP12 | Palatinate | lactating | 29.6.-5.7.2023 | 1 | 49 | 21 |
| SP13 | Palatinate | lactating | 4.7.-14.7.2023 | 3 | 179 | 115 |
| SP14 | Palatinate | lactating | 7.7.-13.7.2023 | 3 | 188 | 120 |
| SP15 | Palatinate | lactating | 10.7.-19.7.2023 | 4 | 212 | 132 |
| SP16 | Palatinate | lactating | 11.7.-19.7.2023 | 3 | 163 | 118 |

**Table S2:** Supporting information on the roosts of the Bechstein´s bats.

| **Roost ID** | **Species** | **Tree condition** | **DBH  (cm)** | **Roost height (m)** | **Distance to next wind turbine (m)** | **X coordinate** | **Y coordinate** |
| --- | --- | --- | --- | --- | --- | --- | --- |
| **Hunsrück HR** | | | | | | | |
| H1 | Oak | vital | 50 | 12 | 230,39 | 344770 | 5514198 |
| H2 | Oak | vital | 60 | 8 | 175,93 | 344822 | 5513848 |
| H3 | Beech | dead | 60 | 10 | 326,86 | 344586 | 5513914 |
| H4 | Beech | vital | 70 |  | 811,07 | 344071 | 5514732 |
| H5 | Beech | dead | 100 | 7 | 811,07 | 344789 | 5514144 |
| H6 | Oak | vital | 50 |  | 177,65 | 344810 | 5513794 |
| H7 | Oak | vital | 50 | 5 | 215,39 | 344082 | 5514360 |
| H8 | Oak | vital | 70 | 6 | 772,68 | 344412 | 5514118 |
| H9 | Oak | vital | 60 | 15 | 501,12 | 344769 | 5514186 |
| H10 | Ash | vital | 100 | 20 | 501,12 | 344334 | 5514613 |
| H11 | Beech | dead | 60 | 6 | 223,33 | 344153 | 5514307 |
| H12 | Oak | vital | 55 | 25 | 223,33 | 344825 | 5514135 |
| H13 | Beech | dead | 70 | 12 | 525,58 | 344368 | 5514533 |
| H14 | Oak | vital | 40 |  | 714,31 | 344334 | 5514523 |
| **Palatinate PL** | | | | | | | |
| P1 | Oak | vital | 50 | 7 | 302,37 | 421596 | 5502500 |
| P2 | Oak | vital | 100 | 15 | 422,09 | 421098 | 5502333 |
| P3 | Erle | vital | 60 | 8 | 445,79 | 421797 | 5502333 |
| P4 | Oak | vital | 50 | 12 | 69,87 | 421014 | 5502691 |
| P5 | Oak | vital | 35 | 17 | 166,42 | 421255 | 5503392 |
| P6 | Oak or Beech | vital |  |  | 281,15 | 420856 | 5502548 |
| P7 | Beech | vital | 70 | 17 | 118,67 | 421218 | 5503355 |
| P8 | Beech | vital | 80 |  | 177,19 | 421329 | 5503276 |
| P9 | Oak | vital | 70 |  | 225,14 | 421210 | 5502909 |
| P10 | Oak | vital | 50 | 7 | 267,41 | 421203 | 5502535 |
| P11 | Oak | vital | 50 |  | 81,83 | 420705 | 5502693 |
| P12 | Oak | vital | 60 | 10 | 274,82 | 420808 | 5502619 |
| P13 | Oak | vital | 45 | 8 | 640,25 | 420302 | 5502506 |
| P14 | Beech | vital | 50 |  | 604,84 | 420428 | 5502551 |
| P15 | Beech | vital | 80 |  | 254,96 | 420283 | 5502894 |
| P16 | Oak | vital | 40 | 12 | 205,66 | 420932 | 5502583 |
| P17 | Beech | vital | 50 | 6 | 174,26 | 420270 | 5502979 |
| P18 | Beech | vital | 30 | 9 | 267,19 | 421707 | 5503069 |
| P19 | Beech | vital | 80 |  | 683,74 | 422101 | 5503364 |
| P20 | Oak | vital | 50 | 25 | 239,77 | 420879 | 5502583 |
| P21 | Oak | vital | 80 |  | 242,16 | 420110 | 5503022 |
| P22 | Oak | vital | 50 | 15 | 154,26 | 421863 | 5502707 |
| P23 | Beech | dead | 65 | 12 | 261,46 | 420860 | 5502572 |
| P24 | Beech | vital | 70 |  | 232,2 | 421870 | 5502952 |
| P25 | Beech | vital | 45 | 8 | 506,3 | 422060 | 5503150 |
| P26 | Beech | vital | 60 | 11 | 378,89 | 421818 | 5503247 |

**Tab. S3a**: Roost use by Bechstein´s bats in Hunsrück in 2019. Number of animals at emergence counts (white lines) and tagged individuals inside the roosts (grey lines) are given for every date and every roost. At some days no emergence counts took place even if the roost was occupied (NA). In some cases not all emerging animals could be seen due to bad weather conditions (+).

| **Roost ID** |  | **30.06.2019** | **01.07.2019** | **02.07.2019** | **03.07.2019** | **04.07.2019** | **05.07.2019** | **06.07.2019** | **07.07.2019** | **08.07.2019** | **09.07.2019** | **10.07.2019** | **11.07.2019** | **12.07.2019** | **13.07.2019** | **14.07.2019** | **15.07.2019** | **16.07.2019** | **17.07.2019** | **18.07.2019** | **19.07.2019** | **20.07.2019** |
| --- | --- | --- | --- | --- | --- | --- | --- | --- | --- | --- | --- | --- | --- | --- | --- | --- | --- | --- | --- | --- | --- | --- |
| H1 |  | 20 | 1 | 0 |  |  |  | 1? |  |  |  |  | 0 |  |  |  |  |  |  |  |  |  |
|  |  | SH1 | SH1 |  |  |  |  |  |  |  |  |  |  |  |  |  |  |  |  |  |  |  |
| H2 |  |  |  | 23 | 22 | 23 | 23 | 22 | 22 | 22 | 21 |  | 0 |  |  |  |  |  |  |  |  |  |
|  |  |  |  | SH1 | SH1 SH2 SH3 | SH1 SH2 SH3 | SH1 SH2 SH3 | SH1 SH2 SH3  SH4 | SH1 SH2 SH3  SH4  SH5 | SH1 SH2 SH3  SH4  SH5 | SH1 SH2 SH3  SH4  SH5 |  |  |  |  |  |  |  |  |  |  |  |
| H3 |  |  |  |  |  |  |  |  |  |  |  | kA | 25 | 26 | 29 | 23 | 21 | 30 | 28 | 0+ |  |  |
|  |  |  |  |  |  |  |  |  |  |  |  | SH2 SH3 SH4 SH5 | SH3 SH4 SH5 SH6 | SH3 SH4 SH5 SH6 | SH3 SH4 SH5 SH6 | SH4 SH5 SH6 | SH4 SH5 SH6 | SH4 SH5 SH6 | SH4 SH5 SH6 | SH4 SH5 SH6 |  |  |
| H4 |  |  |  |  |  |  |  |  |  |  |  |  |  |  |  |  |  | 28 | 38 | 41 | 51 | NA |
|  |  |  |  |  |  |  |  |  |  |  |  |  |  |  |  |  |  | SH7 SH8 | SH7 SH8 | SH7 SH8 | SH7 SH8 | SH7 SH8 |
| H5 |  |  |  |  |  |  |  |  |  |  |  |  |  |  |  |  |  |  |  |  | 29 |  |
|  |  |  |  |  |  |  |  |  |  |  |  |  |  |  |  |  |  |  |  |  | SH4 SH5 SH6 |  |
| H6 |  |  |  |  |  |  |  |  |  |  |  |  |  |  |  |  |  |  |  |  |  | NA |
|  |  |  |  |  |  |  |  |  |  |  |  |  |  |  |  |  |  |  |  |  |  | SH5 SH6 |
| ∑ |  | 20 | 1 | 23 | 22 | 23 | 23 | 23 | 22 | 22 | 21 | kA | 25 | 26 | 29 | 23 | 21 | 58 | 66 | 41 | 80 | kA |

**Tab. S3b**: Roost use by Bechstein´s bats in Hunsrück in 2022. Number of animals at emergence counts (white lines) and tagged individuals inside the roosts (grey lines) are given for every date and every roost. At some days no emergence counts took place even if the roost was occupied (NA). In some cases not all emerging animals could be seen due to bad weather conditions (+).

| **RoostID** | **29.06.2022** | **30.06.2022** | **01.07.2022** | **02.07.2022** | **06.07.2022** | **07.07.2022** | **08.07.2022** | **09.07.2022** | **10.07.2022** | **11.07.2022** | **12.07.2022** | **13.07.2022** | **14.07.2022** | **15.07.2022** | **16.07.2022** | **17.07.2022** |
| --- | --- | --- | --- | --- | --- | --- | --- | --- | --- | --- | --- | --- | --- | --- | --- | --- |
| H4 | 34+ | 19 | 45 | 34-36 | 35+ | NA | NA | 31 | NA | NA |  |  |  |  |  | NA |
|  | SH9  SH10 | SH9 | SH9 | SH9  SH10 | SH11  SH12 | SH11  SH12 | SH11  SH12 | SH11 | SH11  SH12 | SH11 |  |  |  |  |  | SH13 |
| H7 |  | 7 | 0 |  |  |  |  |  |  |  |  |  |  |  |  |  |
|  |  | SH10 |  |  |  |  |  |  |  |  |  |  |  |  |  |  |
| H8 |  |  | 1 |  |  |  |  |  |  |  |  | 29 | kA | kA |  |  |
|  |  |  | SH10 |  |  |  |  |  |  |  |  | SH11  SH13 | SH13 | SH11  SH13 |  |  |
| H9 |  |  |  |  |  |  |  |  | 20 | 23 |  | 17 |  |  |  |  |
|  |  |  |  |  |  |  |  |  | SH13 |  |  | SH14 |  |  |  |  |
| H10 |  |  |  |  |  |  |  |  |  | 65 | kA | 6 |  |  |  |  |
|  |  |  |  |  |  |  |  |  |  | SH12  SH13 | SH11  SH13 |  |  |  |  |  |
| H11 |  |  |  |  |  |  |  |  |  |  |  |  | kA | 1 |  |  |
|  |  |  |  |  |  |  |  |  |  |  |  |  | SH3 |  |  |  |
| H12 |  |  |  |  |  |  |  |  |  |  |  |  | 38 | kA | 35 | kA |
|  |  |  |  |  |  |  |  |  |  |  |  |  | SH14 | SH14 | SH14  SH15 | SH14  SH15 |
| H13 |  |  |  |  |  |  |  |  |  |  |  |  |  |  | kA |  |
|  |  |  |  |  |  |  |  |  |  |  |  |  |  |  | SH13 |  |
| H14 |  |  |  |  |  |  |  |  |  |  |  |  |  |  | kA |  |
|  |  |  |  |  |  |  |  |  |  |  |  |  |  |  | SH11 |  |
| ∑ | 34+ | 26 | 46 | 34-36 | 35+ | kA | kA | 31 | 20 | 88 | kA | 46 | 38 | 1 | 35 | kA |

**Tab. S3c**: Roost use by Bechstein´s bats in Palatinate in 2020. Number of animals at emergence counts (white lines) and tagged individuals inside the roosts (grey lines) are given for every date and every roost. At some days no emergence counts took place even if the roost was occupied (NA). In some cases not all emerging animals could be seen due to bad weather conditions (+).

| **RoostID** | **30.06.2020** | **01.07.2020** | **02.07.2020** | **03.07.2020** | **04.07.2020** | **05.07.2020** | **06.07.2020** | **07.07.2020** | **08.07.2020** | **09.07.2020** | **10.07.2020** | **11.07.2020** | **12.07.2020** | **13.07.2020** | **14.07.2020** | **15.07.2020** | **16.07.2020** | **17.07.2020** | **18.07.2020** | **19.07.2020** | **20.07.2020** | **21.07.2020** | **22.07.2020** | **23.07.2020** | **24.07.2020** | **29.07.2020** |
| --- | --- | --- | --- | --- | --- | --- | --- | --- | --- | --- | --- | --- | --- | --- | --- | --- | --- | --- | --- | --- | --- | --- | --- | --- | --- | --- |
| P1 | 34 | 39 | 40 | 40 | 15+ | 39 | 39 | 35 | kA | kA | 0 |  |  |  |  |  |  |  |  |  |  |  |  |  |  |  |
|  | SP1 | SP1 | SP1 | SP1  SP2 | SP1  SP2 | SP2 | SP2 | SP2 | SP2 | SP2 | 2 |  |  |  |  |  |  |  |  |  |  |  |  |  |  |  |
| P2 |  |  |  |  |  |  |  |  |  | 1 | 1 |  |  |  |  |  |  |  |  |  |  |  |  |  |  |  |
|  |  |  |  |  |  |  |  |  |  | SP3 | SP3 |  |  |  |  |  |  |  |  |  |  |  |  |  |  |  |
| P3 |  |  |  |  |  |  |  |  |  |  |  | 11 | 19 |  |  |  |  |  |  |  |  |  |  |  |  |  |
|  |  |  |  |  |  |  |  |  |  |  |  | SP3 | SP3 |  |  |  |  |  |  |  |  |  |  |  |  |  |
| P4 |  |  |  |  |  |  |  |  |  |  |  |  |  | 21 | 23 | 26 | 28 | 34 | 41 | NA | 17 | 43 | 43 | 58 | NA |  |
|  |  |  |  |  |  |  |  |  |  |  |  |  |  | SP3 | SP3  SP4 | SP3  SP4 | SP3  SP4  SP6 | SP3  SP4  SP6 | SP3  SP4 |  | SP4  SP7 | SP3  SP4  SP5  SP6  SP7  SP8 | SP3  SP4  SP5  SP7  SP8 | SP3  SP4  SP5  SP7  SP8 | SP3  SP7  SP8 |  |
| P5 |  |  |  |  |  |  |  |  |  |  |  |  |  |  |  |  | 1 | 1 | 18 | 10+ |  |  |  |  |  |  |
|  |  |  |  |  |  |  |  |  |  |  |  |  |  |  |  |  | SP5 | SP5 | SP5  SP6 | SP5  SP6 |  |  |  |  |  |  |
| P6 |  |  |  |  |  |  |  |  |  |  |  |  |  |  |  |  |  |  |  | 1+ | 2+ |  |  |  |  |  |
|  |  |  |  |  |  |  |  |  |  |  |  |  |  |  |  |  |  |  |  | SP3 | SP3  SP8 |  |  |  |  |  |
| P7 |  |  |  |  |  |  |  |  |  |  |  |  |  |  |  |  |  |  |  |  | kA | 0 |  |  |  |  |
|  |  |  |  |  |  |  |  |  |  |  |  |  |  |  |  |  |  |  |  |  | SP5  SP6 |  |  |  |  |  |

| **RoostID** | **30.06.2020** | **01.07.2020** | **02.07.2020** | **03.07.2020** | **04.07.2020** | **05.07.2020** | **06.07.2020** | **07.07.2020** | **08.07.2020** | **09.07.2020** | **10.07.2020** | **11.07.2020** | **12.07.2020** | **13.07.2020** | **14.07.2020** | **15.07.2020** | **16.07.2020** | **17.07.2020** | **18.07.2020** | **19.07.2020** | **20.07.2020** | **21.07.2020** | **22.07.2020** | **23.07.2020** | **24.07.2020** | **29.07.2020** |
| --- | --- | --- | --- | --- | --- | --- | --- | --- | --- | --- | --- | --- | --- | --- | --- | --- | --- | --- | --- | --- | --- | --- | --- | --- | --- | --- |
| P8 |  |  |  |  |  |  |  |  |  |  |  |  |  |  |  |  |  |  |  |  |  |  | 0 |  |  |  |
|  |  |  |  |  |  |  |  |  |  |  |  |  |  |  |  |  |  |  |  |  |  |  | SP6 |  |  |  |
| P9 |  |  |  |  |  |  |  |  |  |  |  |  |  |  |  |  |  |  |  |  |  |  |  |  | NA |  |
|  |  |  |  |  |  |  |  |  |  |  |  |  |  |  |  |  |  |  |  |  |  |  |  |  | SP4  SP5 |  |
| P10 |  |  |  |  |  |  |  |  |  |  |  |  |  |  |  |  |  |  |  |  |  |  |  |  |  | NA |
|  |  |  |  |  |  |  |  |  |  |  |  |  |  |  |  |  |  |  |  |  |  |  |  |  |  | SP5 |
| P11 |  |  |  |  |  |  |  |  |  |  |  |  |  |  |  |  |  |  |  |  |  |  |  |  |  | kA |
|  |  |  |  |  |  |  |  |  |  |  |  |  |  |  |  |  |  |  |  |  |  |  |  |  |  | SP7  SP8 |
| ∑ | 34 | 39 | 40 | 40 | 15+ | 39 | 39 | 35 | kA | 1 | 1 | 11 | 19 | 21 | 23 | 26 | 29 | 35 | 59 | 11+ | 19+ | 43 | 43 | 58 | NA | NA |

**Tab. S3d**: Roost use by Bechstein´s bats in Palatinate in 2023. Number of animals at emergence counts (white lines) and tagged individuals inside the roosts (grey lines) are given for every date and every roost. At some days no emergence counts took place even if the roost was occupied (NA). In some cases not all emerging animals could be seen due to bad weather conditions (+).

| **RoostID** | **27.06.2023** | **28.06.2023** | **29.06.2023** | **30.06.2023** | **01.07.2023** | **02.07.2023** | **03.07.2023** | **04.07.2023** | **05.07.2023** | **06.07.2023** | **07.07.2023** | **08.07.2023** | **09.07.2023** | **10.07.2023** | **11.07.2023** | **12.07.2023** | **13.07.2023** | **14.07.2023** | **15.07.2023** | **16.07.2023** | **17.07.2023** | **18.07.2023** | **19.07.2023** |
| --- | --- | --- | --- | --- | --- | --- | --- | --- | --- | --- | --- | --- | --- | --- | --- | --- | --- | --- | --- | --- | --- | --- | --- |
| P12 | 20 | 18 | kA | 24 | 21 | 16 | 22 | 22 | kA | 21 |  | kA | 13 |  |  |  |  |  |  |  |  |  |  |
|  | SP9  SP10 | SP9  SP10 | SP9  SP10  SP11 | SP9  SP10  SP11  SP12 | SP9  SP10  SP11  SP12 | SP9  SP10  SP11  SP12 | SP9  SP10  SP11  SP12 | SP9  SP10  SP11  SP12 | SP9  SP11  SP12 | SP9  SP11 | SP11 | SP11 | SP11  SP14 |  |  |  |  |  |  |  |  |  |  |
| P13 |  |  |  |  |  |  |  |  | 23 | 23 | 23 |  |  |  |  |  |  |  |  |  |  |  |  |
|  |  |  |  |  |  |  |  |  | SP13 | SP13 | SP13 |  |  |  |  |  |  |  |  |  |  |  |  |
| P14 |  |  |  |  |  |  |  |  |  |  |  | 5 |  |  |  |  |  |  |  |  |  |  |  |
|  |  |  |  |  |  |  |  |  |  |  |  | SP13 |  |  |  |  |  |  |  |  |  |  |  |
| P15 |  |  |  |  |  |  |  |  |  |  |  |  | 1 |  |  |  |  |  |  |  |  |  |  |
|  |  |  |  |  |  |  |  |  |  |  |  |  | SP13 |  |  |  |  |  |  |  |  |  |  |
| P16 |  |  |  |  |  |  |  |  |  |  |  |  |  | 5 | 17+ |  |  |  |  |  |  |  |  |
|  |  |  |  |  |  |  |  |  |  |  |  |  |  | SP14 | SP14 |  |  |  |  |  |  |  |  |
| P17 |  |  |  |  |  |  |  |  |  |  |  |  |  | 10 |  |  |  |  |  |  |  |  |  |
|  |  |  |  |  |  |  |  |  |  |  |  |  |  | SP13 | SP13 |  |  |  |  |  |  |  |  |
| P18 |  |  |  |  |  |  |  |  |  |  |  |  |  |  | 1+ | 8 |  |  |  | kA |  |  |  |
|  |  |  |  |  |  |  |  |  |  |  |  |  |  |  | SP15 | SP15 |  |  |  | SP15 |  |  |  |
| P19 |  |  |  |  |  |  |  |  |  |  |  |  |  |  |  | kA | kA |  |  |  |  |  |  |
|  |  |  |  |  |  |  |  |  |  |  |  |  |  |  |  | SP14 | SP14 |  |  |  |  |  |  |
| P20 |  |  |  |  |  |  |  |  |  |  |  |  |  |  |  | 8 |  |  | kA |  |  |  |  |
|  |  |  |  |  |  |  |  |  |  |  |  |  |  |  |  | SP16 |  |  | SP15  SP16 |  |  |  |  |
| P21 |  |  |  |  |  |  |  |  |  |  |  |  |  |  |  | NA | NA | NA |  |  |  |  |  |
|  |  |  |  |  |  |  |  |  |  |  |  |  |  |  |  | SP13 | SP13 | SP13 |  |  |  |  |  |
| P22 |  |  |  |  |  |  |  |  |  |  |  |  |  |  |  |  | 24 | NA |  |  |  |  |  |
|  |  |  |  |  |  |  |  |  |  |  |  |  |  |  |  |  | SP15  SP16 | SP15  SP16 |  |  |  |  |  |
| P23 |  |  |  |  |  |  |  |  |  |  |  |  |  |  |  |  |  | 30 |  |  |  |  |  |
|  |  |  |  |  |  |  |  |  |  |  |  |  |  |  |  |  |  | SP14 |  |  |  |  |  |
| P24 |  |  |  |  |  |  |  |  |  |  |  |  |  |  |  |  |  |  |  | 1+ |  |  |  |
|  |  |  |  |  |  |  |  |  |  |  |  |  |  |  |  |  |  |  |  | SP16 |  |  |  |
| P25 |  |  |  |  |  |  |  |  |  |  |  |  |  |  |  |  |  |  |  |  | 16 | NA | NA |
|  |  |  |  |  |  |  |  |  |  |  |  |  |  |  |  |  |  |  |  |  | SP15  SP16 | SP15  SP16 | SP15 |
| P26 |  |  |  |  |  |  |  |  |  |  |  |  |  |  |  |  |  |  |  |  |  |  | NA |
|  |  |  |  |  |  |  |  |  |  |  |  |  |  |  |  |  |  |  |  |  |  |  | SP16 |
| ∑ | 20 | 18 | kA | 24 | 21 | 16 | 22 | 22 | 23 | 44 | 23 | 5 | 14 | 15 | 18+ | 16 | 24 | 30 | kA | 1+ | 16 | NA | NA |

**Tab. S4:** Size of individual MCP (home range) and size and number of individual 95% LoCoH (foraging search areas) and 50 %-LoCoH (core foraging areas) in both study areas.

| **IndividualID** | **MCP (ha)** | **95 % LoCoH [ha]** | **Number 95 % LoCoH** | **50 % LoCoH [ha]** | **Number 50 % LoCoH** |
| --- | --- | --- | --- | --- | --- |
| **Hunsrück** | | | | | |
| SH1 | 52,26 | 10,21 | 2 | 2,27 | 1 |
| SH2 | 6,59 | 2,75 | 1 | 0,55 | 1 |
| SH3 | 7,00 | 2,14 | 1 | 0,49 | 1 |
| SH4 | 7,52 | 2,79 | 1 | 0,87 | 1 |
| SH5 | 53,88 | 17,58 | 1 | 5,90 | 1 |
| SH6 | 12,16 | 5,14 | 1 | 0,73 | 1 |
| SH7 | 172,05 | 11,69 | 2 | - |  |
| SH8 | 19,15 | 9,93 | 1 | 2,27 | 1 |
| SH9 | 20,07 | 4,68 | 1 | 0,87 | 1 |
| SH10 | 8,60 | 4,05 | 1 | 0,82 | 1 |
| SH11 | 24,78 | 6,13 | 1 | 0,86 | 1 |
| SH12 | 56,27 | 24,47 | 1 | 2,15 | 1 |
| SH13 | 99,68 | 32,16 | 2 | 5,89 | 1 |
| SH14 | 34,42 | 5,69 | 2 | 1,43 | 1 |
| SH15 | 31,02 | 4,30 | 2 | 0,74 | 1 |
| **Pfalz** | | | | | |
| SP1 | 92,27 | 25,81 | 2 | 5,09 | 2 |
| SP2 | 26,70 | 12,23 | 1 | 2,52 | 1 |
| SP3 | 35,83 | 13,13 | 1 | 2,31 | 1 |
| SP4 | 65,56 | 15,20 | 2 | 2,08 | 1 |
| SP5 | 51,09 | 12,92 | 1 | 1,44 | 1 |
| SP6 | 51,67 | 18,11 | 1 | 2,19 | 1 |
| SP7 | 77,71 | 32,20 | 3 | 6,95 | 2 |
| SP8 | 17,15 | 8,69 | 1 | 1,79 | 1 |
| SP9 | 10,22 | 4,30 | 1 | 0,87 | 1 |
| SP10 | 15,97 | 6,54 | 1 | 0,94 | 1 |
| SP11 | 31,01 | 13,76 | 2 | 2,67 | 1 |
| SP12 | 15,38 | 7,68 | 1 | - |  |
| SP13 | 28,72 | 13,15 | 1 | 3,51 | 1 |
| SP14 | 19,34 | 7,98 | 1 | 2,09 | 1 |
| SP15 | 48,48 | 18,25 | 1 | 3,94 | 1 |
| SP16 | 33,34 | 16,37 | 1 | 3,31 | 1 |
