# Supplementary material for: Habitat use of Bechstein´s bats (Myotis bechsteinii) within wind parks in forests
Source: PLoS One. 2026 Mar 25;21(3):e0344730. doi: 10.1371/journal.pone.0344730 (PMC13016307; doi:10.1371/journal.pone.0344730)
Supplement: S2 File — (HTML) [file pone.0344730.s002.html]

R-code of the analyses for the manuscript Habitat use of Bechstein´s bats (Myotis bechsteinii) within wind parks in forests


Code 

- Show All Code
- Hide All Code

# R-code of the analyses for the manuscript Habitat use of Bechstein´s bats (Myotis bechsteinii) within wind parks in forests

#### Johanna Hurst and Fränzi Korner-Nievergelt

#### 2025-11-19

# 1 Conditional logistic regression to describe habitat selection

Within each individual home-range, we assigned to each bat location an absence point that we randomly chose from a 100x100m grid of points where habitat variables have been measured in the field. Thus the response variable was the binary variable use (0/1).
To assess the sensitivity towards the random assignment of the absence points, we created 10 data sets, each with a different set of randomly chosen absence points and we fitted the model to all 10 data sets.

## 1.1 Correlation matrix among the habitat variables

```
load("data/ppdat_for_public.rda") # original data file

cor(ppdat[,c("dist2wea_2",
                      "Kraut.num.z", 
                      "anteil_laubbaum.z", "Strauch_Ge.num.z", 
                      "Baum.num.z",  "median_bhd.z",  "sd_bhd.z")], 
    use="pairwise.complete.obs")
```

```
##                    dist2wea_2 Kraut.num.z anteil_laubbaum.z Strauch_Ge.num.z
## dist2wea_2         1.00000000  0.09652213       -0.09163577       0.01302769
## Kraut.num.z        0.09652213  1.00000000       -0.25661199       0.12686680
## anteil_laubbaum.z -0.09163577 -0.25661199        1.00000000       0.11209378
## Strauch_Ge.num.z   0.01302769  0.12686680        0.11209378       1.00000000
## Baum.num.z        -0.04393476 -0.35013352       -0.05097908      -0.40206523
## median_bhd.z      -0.05116171  0.04251231       -0.05908852      -0.39907784
## sd_bhd.z           0.02054706  0.05863553        0.11781417      -0.20171900
##                    Baum.num.z median_bhd.z    sd_bhd.z
## dist2wea_2        -0.04393476  -0.05116171  0.02054706
## Kraut.num.z       -0.35013352   0.04251231  0.05863553
## anteil_laubbaum.z -0.05097908  -0.05908852  0.11781417
## Strauch_Ge.num.z  -0.40206523  -0.39907784 -0.20171900
## Baum.num.z         1.00000000   0.24328730  0.17978287
## median_bhd.z       0.24328730   1.00000000  0.38998382
## sd_bhd.z           0.17978287   0.38998382  1.00000000
```

## 1.2 Model fit

```
library(rstanarm)


for(kk in 1:10){
load(paste0("data/dat", kk,"_neu301224.rda"))
dat <- dat[!is.na(dat$datetime),]
dat <- dat[order(dat$pairID),]
dat$dist2quart.z <- as.numeric(scale(dat$dist2quart))

# Chose every 3 observation to reduce temporal correlation and increase model fitting time
dagg <- aggregate(datetime~pairID, dat, FUN=function(x) unique(x))
dagg$indIDyear <-aggregate(indIDyear~pairID, dat, FUN=function(x) unique(x))$indIDyear
dagg$date.date <- strptime(dagg$datetime, format="%Y-%m-%d %H:%M:%S")
dagg <- dagg[!is.na(dagg$date.date),]
dagg <- dagg[order(dagg$indIDyear, dagg$date.date),]
dagg <- dagg[seq(1,nrow(dagg), by=3),]

datred <- dat[is.element(dat$pairID, dagg$pairID),]
table(datred$gebiet, datred$use)


datred <- datred[order(datred$pairID),]
cat("Daten", kk,":", table(dat$use)[2], "\n")


mod2 <- stan_clogit(use ~ dist2wea_2.z + dist2quart.z +
                      Kraut.num.z + 
                      anteil_laubbaum.z + Strauch_Ge.num.z + 
                      Baum.num.z + median_bhd.z + 
                      sd_bhd.z + 
                      dist2wea_2.z:(#rotor_speed.z + wind.z + 
                                    dist2quart.z + 
                                      Kraut.num.z +
                                    anteil_laubbaum.z + 
                                    Strauch_Ge.num.z + 
                                    Baum.num.z + median_bhd.z + 
                                    sd_bhd.z) + 
                      (dist2wea_2.z + dist2quart.z +
                         anteil_laubbaum.z + Strauch_Ge.num.z  + Baum.num.z + median_bhd.z + sd_bhd.z -1|indIDyear), strata=pairID,
  data= datred)

save(mod2, file=paste0("modelfits/mod2b_dat_ohnefehlendeWEA", kk,".rda"))
}
```

```
# check convergence and MCMC performance
load("modelfits/mod140125_1.rda")
par(mfrow=c(1,2))
hist(summary(mod2)[,"Rhat"])
hist(summary(mod2)[,"n_eff"])
```

## 1.3 Check correlation structure

Figure 1.1: Temporal and spatial correlation structures of the residuals in the model (first data set used).

```
## Warning: Paket 'colourvalues' wurde unter R Version 4.2.3 erstellt
## Lade nötiges Paket: spam
## Spam version 2.9-1 (2022-08-07) is loaded.
## Type 'help( Spam)' or 'demo( spam)' for a short introduction 
## and overview of this package.
## Help for individual functions is also obtained by adding the
## suffix '.spam' to the function name, e.g. 'help( chol.spam)'.
## 
## Attache Paket: 'spam'
## Die folgenden Objekte sind maskiert von 'package:base':
## 
##     backsolve, forwardsolve
## Lade nötiges Paket: viridis
## Warning: Paket 'viridis' wurde unter R Version 4.2.3 erstellt
## Lade nötiges Paket: viridisLite
## Warning: Paket 'viridisLite' wurde unter R Version 4.2.3 erstellt
## 
## Try help(fields) to get started.
## Warning: Paket 'sf' wurde unter R Version 4.2.3 erstellt
## Linking to GEOS 3.9.3, GDAL 3.5.2, PROJ 8.2.1; sf_use_s2() is TRUE
```

Figure 1.2: Temporal and spatial correlation structures of the residuals in the model (first data set used).

We see both slight temporal and spatial correlation in the residuals. As a consequence, we will be overconfident in estimated individual effects (i.e. within-individual effects) due to pseudo-replication within individuals. However, as we are interested in average effects, averaged over individuals, and we account for within individual correlation by individual-specific effect sizes (random slopes), the temporal and spatial correlation in our data will not noticably affect our results. I.e., the credible intervals reported in our study are primarily determined by the number of individuals rather than by the (slightly pseudo-replicated) number of locations per individual.

## 1.4 Results

```
load("data/ppdat_for_public.rda") # original data file
load("modelfits/mod140125_1.rda")
bsim1 <- as.data.frame(mod2)
load("modelfits/mod140125_2.rda")
bsim1_d2 <- as.data.frame(mod2)
load("modelfits/mod140125_3.rda")
bsim1_d3 <- as.data.frame(mod2)
load("modelfits/mod140125_4.rda")
bsim1_d4 <- as.data.frame(mod2)
load("modelfits/mod140125_5.rda")
bsim1_d5 <- as.data.frame(mod2)
load("modelfits/mod140125_6.rda")
bsim1_d6 <- as.data.frame(mod2)
load("modelfits/mod140125_7.rda")
bsim1_d7 <- as.data.frame(mod2)
load("modelfits/mod140125_8.rda")
bsim1_d8 <- as.data.frame(mod2)
load("modelfits/mod140125_9.rda")
bsim1_d9 <- as.data.frame(mod2)
load("modelfits/mod140125_10.rda")
bsim1_d10 <- as.data.frame(mod2)

bsim <- merge(bsim1, bsim1_d2, all=TRUE)
bsim <- merge(bsim, bsim1_d3, all=TRUE)
bsim <- merge(bsim, bsim1_d4, all=TRUE)
bsim <- merge(bsim, bsim1_d5, all=TRUE)
bsim <- merge(bsim, bsim1_d6, all=TRUE)
bsim <- merge(bsim, bsim1_d7, all=TRUE)
bsim <- merge(bsim, bsim1_d8, all=TRUE)
bsim <- merge(bsim, bsim1_d9, all=TRUE)
bsim <- merge(bsim, bsim1_d10, all=TRUE)


ndatasets <- 10

tab <- matrix(ncol=3, nrow=ncol(bsim))
tab[,1] <- apply(bsim, 2, mean)
tab[,2] <- apply(bsim, 2, quantile, probs=0.025)
tab[,3] <- apply(bsim, 2, quantile, probs=0.975)
colnames(tab) <- c("Estimate", "lwr95", "upr95")
rownames(tab) <- names(bsim)
tab <- tab[c(1:15, 233, 240, 246, 251, 255,258, 260),]
rownames(tab)[16:22] <- c("Among-ind SD dist2wea.z", "Among-ind SD dist2quart.z", "Among-ind SD anteil_laubbaum.z", "Among-ind SD Strauch_Ge.num.z", "Among-ind SD Baum.num.z", "Among-ind median_bhd.z", "Among-ind SD sd_bhd.z")
tab[16:22,1:3] <- sqrt(tab[16:22,1:3]) # transform variance into SD 
  
  
library(knitr)
kable(tab, dig=2, caption="Summay of the posterior distributions of the model parameters which includes the variance inherent in the sensitivity analyses towards random assignment of the absence points.")
```

Table 1.1: Summay of the posterior distributions of the model parameters which includes the variance inherent in the sensitivity analyses towards random assignment of the absence points.

|  | Estimate | lwr95 | upr95 |
| --- | --- | --- | --- |
| dist2wea\_2.z | -0.97 | -1.78 | -0.25 |
| dist2quart.z | -3.25 | -4.80 | -2.13 |
| Kraut.num.z | -0.14 | -0.45 | 0.14 |
| anteil\_laubbaum.z | 0.17 | -0.33 | 0.71 |
| Strauch\_Ge.num.z | -0.04 | -0.52 | 0.38 |
| Baum.num.z | 0.07 | -0.37 | 0.52 |
| median\_bhd.z | -0.09 | -0.52 | 0.33 |
| sd\_bhd.z | 0.60 | 0.10 | 1.25 |
| dist2wea\_2.z:dist2quart.z | 0.94 | 0.50 | 1.42 |
| dist2wea\_2.z:Kraut.num.z | 0.18 | -0.19 | 0.52 |
| dist2wea\_2.z:anteil\_laubbaum.z | -0.10 | -0.45 | 0.24 |
| dist2wea\_2.z:Strauch\_Ge.num.z | 0.09 | -0.19 | 0.37 |
| dist2wea\_2.z:Baum.num.z | -0.12 | -0.59 | 0.27 |
| dist2wea\_2.z:median\_bhd.z | -0.16 | -0.48 | 0.17 |
| dist2wea\_2.z:sd\_bhd.z | -0.04 | -0.37 | 0.30 |
| Among-ind SD dist2wea.z | 1.61 | 0.88 | 2.40 |
| Among-ind SD dist2quart.z | 2.46 | 1.51 | 3.78 |
| Among-ind SD anteil\_laubbaum.z | 0.90 | 0.29 | 1.55 |
| Among-ind SD Strauch\_Ge.num.z | 0.65 | 0.27 | 1.07 |
| Among-ind SD Baum.num.z | 0.61 | 0.28 | 0.97 |
| Among-ind median\_bhd.z | 0.62 | 0.21 | 1.08 |
| Among-ind SD sd\_bhd.z | 0.74 | 0.27 | 1.29 |

```
maxy <- 5

# Einfluss Distanz zu WEA
variables <- c("dist2quart.z", "anteil_laubbaum.z", "Strauch_Ge.num.z", "median_bhd.z", "sd_bhd.z", "Baum.num.z", "Kraut.num.z")

newdat <- data.frame(dist2wea=seq(0, 1250, length=100))
newdat$dist2wea.z <- (newdat$dist2wea-mean(ppdat$dist2wea))/sd(ppdat$dist2wea)
xmat <- model.matrix(~dist2wea.z, data=newdat)

for(v in variables){
fitmat <- matrix(nrow=nrow(newdat), ncol=nrow(bsim1))
fitmat_d2 <- matrix(nrow=nrow(newdat), ncol=nrow(bsim1_d2))
fitmat_d3 <- matrix(nrow=nrow(newdat), ncol=nrow(bsim1_d3))
fitmat_d4 <- matrix(nrow=nrow(newdat), ncol=nrow(bsim1_d4))
fitmat_d5 <- matrix(nrow=nrow(newdat), ncol=nrow(bsim1_d5))
fitmat_d6 <- matrix(nrow=nrow(newdat), ncol=nrow(bsim1_d6))
fitmat_d7 <- matrix(nrow=nrow(newdat), ncol=nrow(bsim1_d7))
fitmat_d8 <- matrix(nrow=nrow(newdat), ncol=nrow(bsim1_d8))
fitmat_d9 <- matrix(nrow=nrow(newdat), ncol=nrow(bsim1_d9))
fitmat_d10 <- matrix(nrow=nrow(newdat), ncol=nrow(bsim1_d10))

for(i in 1:nrow(bsim1)) {
  fitmat[,i] <- xmat%*%as.numeric(bsim1[i,c(v, paste0("dist2wea_2.z:",v))])
  fitmat_d2[,i] <- xmat%*%as.numeric(bsim1_d2[i,c(v, paste0("dist2wea_2.z:",v))])
  fitmat_d3[,i] <- xmat%*%as.numeric(bsim1_d3[i,c(v, paste0("dist2wea_2.z:",v))])
  fitmat_d4[,i] <- xmat%*%as.numeric(bsim1_d4[i,c(v, paste0("dist2wea_2.z:",v))])
  fitmat_d5[,i] <- xmat%*%as.numeric(bsim1_d5[i,c(v, paste0("dist2wea_2.z:",v))])
  fitmat_d6[,i] <- xmat%*%as.numeric(bsim1_d6[i,c(v, paste0("dist2wea_2.z:",v))])
  fitmat_d7[,i] <- xmat%*%as.numeric(bsim1_d7[i,c(v, paste0("dist2wea_2.z:",v))])
  fitmat_d8[,i] <- xmat%*%as.numeric(bsim1_d8[i,c(v, paste0("dist2wea_2.z:",v))])
  fitmat_d9[,i] <- xmat%*%as.numeric(bsim1_d9[i,c(v, paste0("dist2wea_2.z:",v))])
  fitmat_d10[,i] <- xmat%*%as.numeric(bsim1_d10[i,c(v, paste0("dist2wea_2.z:",v))])
    }

eval(parse(text=paste0("newdat$fit_d1", v, "<- apply(fitmat, 1, mean)")))
eval(parse(text=paste0("newdat$lwr_d1", v, "<- apply(fitmat, 1, quantile, probs=0.025)")))
eval(parse(text=paste0("newdat$upr_d1", v, "<- apply(fitmat, 1, quantile, probs=0.975)")))

 eval(parse(text=paste0("newdat$fit_d2", v, "<- apply(fitmat_d2, 1, mean)")))
 eval(parse(text=paste0("newdat$lwr_d2", v, "<- apply(fitmat_d2, 1, quantile, probs=0.025)")))
 eval(parse(text=paste0("newdat$upr_d2", v, "<- apply(fitmat_d2, 1, quantile, probs=0.975)")))

  eval(parse(text=paste0("newdat$fit_d3", v, "<- apply(fitmat_d3, 1, mean)")))
 eval(parse(text=paste0("newdat$lwr_d3", v, "<- apply(fitmat_d3, 1, quantile, probs=0.025)")))
 eval(parse(text=paste0("newdat$upr_d3", v, "<- apply(fitmat_d3, 1, quantile, probs=0.975)")))

   eval(parse(text=paste0("newdat$fit_d4", v, "<- apply(fitmat_d4, 1, mean)")))
 eval(parse(text=paste0("newdat$lwr_d4", v, "<- apply(fitmat_d4, 1, quantile, probs=0.025)")))
 eval(parse(text=paste0("newdat$upr_d4", v, "<- apply(fitmat_d4, 1, quantile, probs=0.975)")))

    eval(parse(text=paste0("newdat$fit_d5", v, "<- apply(fitmat_d5, 1, mean)")))
 eval(parse(text=paste0("newdat$lwr_d5", v, "<- apply(fitmat_d5, 1, quantile, probs=0.025)")))
 eval(parse(text=paste0("newdat$upr_d5", v, "<- apply(fitmat_d5, 1, quantile, probs=0.975)")))

    eval(parse(text=paste0("newdat$fit_d6", v, "<- apply(fitmat_d6, 1, mean)")))
 eval(parse(text=paste0("newdat$lwr_d6", v, "<- apply(fitmat_d6, 1, quantile, probs=0.025)")))
 eval(parse(text=paste0("newdat$upr_d6", v, "<- apply(fitmat_d6, 1, quantile, probs=0.975)")))

     eval(parse(text=paste0("newdat$fit_d7", v, "<- apply(fitmat_d7, 1, mean)")))
 eval(parse(text=paste0("newdat$lwr_d7", v, "<- apply(fitmat_d7, 1, quantile, probs=0.025)")))
 eval(parse(text=paste0("newdat$upr_d7", v, "<- apply(fitmat_d7, 1, quantile, probs=0.975)")))

     eval(parse(text=paste0("newdat$fit_d8", v, "<- apply(fitmat_d8, 1, mean)")))
 eval(parse(text=paste0("newdat$lwr_d8", v, "<- apply(fitmat_d8, 1, quantile, probs=0.025)")))
 eval(parse(text=paste0("newdat$upr_d8", v, "<- apply(fitmat_d8, 1, quantile, probs=0.975)")))

     eval(parse(text=paste0("newdat$fit_d9", v, "<- apply(fitmat_d9, 1, mean)")))
 eval(parse(text=paste0("newdat$lwr_d9", v, "<- apply(fitmat_d9, 1, quantile, probs=0.025)")))
 eval(parse(text=paste0("newdat$upr_d9", v, "<- apply(fitmat_d9, 1, quantile, probs=0.975)")))

     eval(parse(text=paste0("newdat$fit_d10", v, "<- apply(fitmat_d10, 1, mean)")))
 eval(parse(text=paste0("newdat$lwr_d10", v, "<- apply(fitmat_d10, 1, quantile, probs=0.025)")))
 eval(parse(text=paste0("newdat$upr_d10", v, "<- apply(fitmat_d10, 1, quantile, probs=0.975)")))

}


colpolygon <- scales::alpha("deepskyblue4", 0.2)
colline <- "black"
cm <- 1.5 # cex.main

par(mfrow=c(4,2), mar=c(1,1,2,0.1), oma=c(3,2,0,0), mgp=c(1.5,0.3,0), tck=0.02, cex.axis=1.2, cex.lab=1.5)
plot(newdat$dist2wea, exp(newdat$fit_d1anteil_laubbaum.z), ylim=c(0, maxy), type="n", xlab=NA, xaxt="n", ylab=NA, main="Proportion of broad-leafed trees", las=1, cex.main=cm) # Anteil Laubbaum

abline(h=1, col="grey")

for(d in 1:ndatasets){
  di <- paste0("d", d)
polygon(c(newdat$dist2wea, rev(newdat$dist2wea)),
        exp(c(newdat[,paste0("lwr_", di, "anteil_laubbaum.z")], rev(newdat[,paste0("upr_", di, "anteil_laubbaum.z")]))), 
        col=colpolygon, border=NA)
}
indexfit <- grep("fit", names(newdat))
indexvar <- grep("anteil_laubbaum", names(newdat))
ii <- indexvar[is.element(indexvar, indexfit)]
fit <- apply(newdat[,ii], 1, mean)
  lines(newdat$dist2wea, exp(fit), lwd=2, col=colline)

text(min(newdat$dist2wea), maxy, "A", adj=c(0,1), cex=1.5)


plot(newdat$dist2wea, exp(newdat$fit_d1Strauch_Ge.num.z), ylim=c(0, maxy), type="n", xlab=NA, xaxt="n", ylab=NA, main="Shrub layer coverage", yaxt="n", cex.main=cm) #Strauchschicht gesamt
abline(h=1, col="grey")

for(d in 1:ndatasets){
  di <- paste0("d", d)
polygon(c(newdat$dist2wea, rev(newdat$dist2wea)),
        exp(c(newdat[,paste0("lwr_", di, "Strauch_Ge.num.z")], rev(newdat[,paste0("upr_", di, "Strauch_Ge.num.z")]))), 
        col=colpolygon, border=NA)
}
indexfit <- grep("fit", names(newdat))
indexvar <- grep("Strauch_Ge.num", names(newdat))
ii <- indexvar[is.element(indexvar, indexfit)]
fit <- apply(newdat[,ii], 1, mean)
  lines(newdat$dist2wea, exp(fit), lwd=2, col=colline)

 text(min(newdat$dist2wea), maxy, "B", adj=c(0,1), cex=1.5)

plot(newdat$dist2wea, exp(newdat$fit_d1dist2quart.z), ylim=c(0, maxy), type="n", xlab="", xaxt="n", ylab=NA, main="Distance to roost", las=1, cex.main=cm) # Distanz zu Quartier
abline(h=1, col="grey")
for(d in 1:ndatasets){
  di <- paste0("d", d)
polygon(c(newdat$dist2wea, rev(newdat$dist2wea)),
        exp(c(newdat[,paste0("lwr_", di, "dist2quart.z")], rev(newdat[,paste0("upr_", di, "dist2quart.z")]))), 
        col=colpolygon, border=NA)
}
indexfit <- grep("fit", names(newdat))
indexvar <- grep("dist2quart", names(newdat))
ii <- indexvar[is.element(indexvar, indexfit)]
fit <- apply(newdat[,ii], 1, mean)
  lines(newdat$dist2wea, exp(fit), lwd=2, col=colline)

  
text(min(newdat$dist2wea), maxy, "C", adj=c(0,1), cex=1.5)


plot(newdat$dist2wea, exp(newdat$fit_d1Baum.num.z), ylim=c(0, maxy), type="l", xlab="", xaxt="n", ylab="", main="Tree layer coverage", yaxt="n", cex.main=cm) # Baumkronendeckung
abline(h=1, col="grey")
for(d in 1:ndatasets){
  di <- paste0("d", d)
polygon(c(newdat$dist2wea, rev(newdat$dist2wea)),
        exp(c(newdat[,paste0("lwr_", di, "Baum.num.z")], rev(newdat[,paste0("upr_", di, "Baum.num.z")]))), 
        col=colpolygon, border=NA)
}
indexfit <- grep("fit", names(newdat))
indexvar <- grep("Baum.num", names(newdat))
ii <- indexvar[is.element(indexvar, indexfit)]
fit <- apply(newdat[,ii], 1, mean)
  lines(newdat$dist2wea, exp(fit), lwd=2, col=colline)

  
text(min(newdat$dist2wea), maxy, "D", adj=c(0,1), cex=1.5)

  
plot(newdat$dist2wea, exp(newdat$fit_d1median_bhd.z), ylim=c(0, maxy), type="n", xlab="", xaxt="n", ylab="", main="Median of DBH", las=1, cex.main=cm) #Median BHD
abline(h=1, col="grey")

for(d in 1:ndatasets){
  di <- paste0("d", d)
polygon(c(newdat$dist2wea, rev(newdat$dist2wea)),
        exp(c(newdat[,paste0("lwr_", di, "median_bhd.z")], rev(newdat[,paste0("upr_", di, "median_bhd.z")]))), 
        col=colpolygon, border=NA)
}
indexfit <- grep("fit", names(newdat))
indexvar <- grep("median_bhd", names(newdat))
ii <- indexvar[is.element(indexvar, indexfit)]
fit <- apply(newdat[,ii], 1, mean)
  lines(newdat$dist2wea, exp(fit), lwd=2, col=colline)

text(min(newdat$dist2wea), maxy, "E", adj=c(0,1), cex=1.5)

 
plot(newdat$dist2wea, exp(newdat$fit_d1sd_bhd.z), ylim=c(0, maxy), type="n", xlab=, ylab="", main="Standard deviation of DBH", yaxt="n", cex.main=cm)
mtext("Distance to wind turbine [m]", side=1, line=2)
abline(h=1, col="grey")

for(d in 1:ndatasets){
  di <- paste0("d", d)
polygon(c(newdat$dist2wea, rev(newdat$dist2wea)),
        exp(c(newdat[,paste0("lwr_", di, "sd_bhd.z")], rev(newdat[,paste0("upr_", di, "sd_bhd.z")]))), 
        col=colpolygon, border=NA)
}
indexfit <- grep("fit", names(newdat))
indexvar <- grep("sd_bhd", names(newdat))
ii <- indexvar[is.element(indexvar, indexfit)]
fit <- apply(newdat[,ii], 1, mean)
  lines(newdat$dist2wea, exp(fit), lwd=2, col=colline)
  
  
text(min(newdat$dist2wea), maxy, "F", adj=c(0,1), cex=1.5)

  
plot(newdat$dist2wea, exp(newdat$fit_d1Kraut.num.z), ylim=c(0, maxy), type="n", xlab="", ylab="", main="Herb layer coverage", las=1, cex.main=cm) # Krautschicht
abline(h=1, col="grey")
mtext("Distance to wind turbine [m]", side=1, line=2)
for(d in 1:ndatasets){
  di <- paste0("d", d)
polygon(c(newdat$dist2wea, rev(newdat$dist2wea)),
        exp(c(newdat[,paste0("lwr_", di, "Kraut.num.z")], rev(newdat[,paste0("upr_", di, "Kraut.num.z")]))),
        col=colpolygon, border=NA)
}
indexfit <- grep("fit", names(newdat))
indexvar <- grep("Kraut.num.z", names(newdat))
ii <- indexvar[is.element(indexvar, indexfit)]
fit <- apply(newdat[,ii], 1, mean)
  lines(newdat$dist2wea, exp(fit), lwd=2, col=colline)

text(min(newdat$dist2wea), maxy, "G", adj=c(0,1), cex=1.5)


mtext("Preference", outer=TRUE, side=2, line=0.5)
#dev.off()
```

Figure 1.3: Fig. 6

# 2 Distance to the wind turbine in relation to wind and rotor speed

## 2.1 Model fit

```
library(arm)
load("data/dat1_neu301224.rda")
datd <- dat[dat$use==1,]
datd$dist2quart.z <- as.numeric(scale(datd$dist2quart))
md <- lmer(dist2wea_2~ wind.z + rotor_speed.z + dist2quart.z +
             gebiet +
             wind.z:rotor_speed.z +
             wind.z:gebiet + rotor_speed.z:gebiet +
             rotor_speed.z:gebiet:wind.z +
             (1|individual),
           data=datd)
save(md, file="modelfits/model_distanz.rda")
```

```
load("modelfits/model_distanz.rda")
plot(md)
```

```
qqnorm(resid(md))
qqline(resid(md))
```

## 2.2 Results

```
library(arm)
```

```
## Lade nötiges Paket: MASS
```

```
## Lade nötiges Paket: Matrix
```

```
## Warning: Paket 'Matrix' wurde unter R Version 4.2.3 erstellt
```

```
## 
## Attache Paket: 'Matrix'
```

```
## Das folgende Objekt ist maskiert 'package:spam':
## 
##     det
```

```
## Lade nötiges Paket: lme4
```

```
## 
## arm (Version 1.13-1, built: 2022-8-25)
```

```
## Working directory is C:/Users/fk/oikostat Dropbox/Team-Ordner_oikostat/Projekte/Frinat/Habitatpraeferenz Bechsteinfledermaus/analysen
```

```
## 
## Attache Paket: 'arm'
```

```
## Das folgende Objekt ist maskiert 'package:spam':
## 
##     display
```

```
load("data/dat1_neu301224.rda")
datd <- dat[dat$use==1,]

nsim <- 5000
bsim <- sim(md, n.sim=nsim)

tab <- matrix(ncol=3, nrow=ncol(bsim@fixef))
tab[,1] <- apply(bsim@fixef, 2, mean)
tab[,2] <- apply(bsim@fixef, 2, quantile, probs=0.025)
tab[,3] <- apply(bsim@fixef, 2, quantile, probs=0.975)
colnames(tab) <- c("Estimate", "lwr95", "upr95")
rownames(tab) <- c("(Intercept)", "wind.z", "rotor_speed.z", "dist2quart.z", "gebietPalatinate", "wind.z:rotor_speed.z", "wind.z:gebietPalatinate", "rotor_speed.z:gebietPalatinate", "wind.z:rotor_speed.z:gebietPalatinate") # colnames(bsim@fixef), original data contains name of wind park that should not be public

kable(tab, dig=2, caption="Summary of the posterior distributions of the model parameters.")
```

Table 2.1: Summary of the posterior distributions of the model parameters.

|  | Estimate | lwr95 | upr95 |
| --- | --- | --- | --- |
| (Intercept) | 429.23 | 365.61 | 495.01 |
| wind.z | -2.18 | -18.73 | 14.92 |
| rotor\_speed.z | -38.43 | -52.12 | -24.60 |
| dist2quart.z | 29.57 | 24.22 | 35.11 |
| gebietPalatinate | -236.60 | -261.51 | -210.59 |
| wind.z:rotor\_speed.z | 64.27 | 52.86 | 75.64 |
| wind.z:gebietPalatinate | 12.81 | -6.72 | 32.09 |
| rotor\_speed.z:gebietPalatinate | 5.42 | -16.79 | 27.52 |
| wind.z:rotor\_speed.z:gebietPalatinate | -39.11 | -53.29 | -24.89 |

```
tt <- data.frame(Variable=c("Wind", "Rotor speed", "Distance to roost"),
                 mean=NA, SD=NA)
tt$mean[1] <- mean(ppdat$wind, na.rm=TRUE)
tt$mean[2] <- mean(ppdat$rotor_speed, na.rm=TRUE)
tt$mean[3] <- mean(datd$dist2quart)

tt$SD[1] <- sd(ppdat$wind, na.rm=TRUE)
tt$SD[2] <- sd(ppdat$rotor_speed, na.rm=TRUE)
tt$SD[3] <- sd(datd$dist2quart)
kable(tt, dig=1, caption="Standard deviations of the numeric predictor variables used in the model.")
```

Table 2.2: Standard deviations of the numeric predictor variables used in the model.

| Variable | mean | SD |
| --- | --- | --- |
| Wind | 5.0 | 2.4 |
| Rotor speed | 5.5 | 5.2 |
| Distance to roost | 523.3 | 467.3 |

Reading example: At an average wind speed (5 m/s) the distance to the WEA decreases in average by 38.3 m when rotor speed increases by 5.2 (=1 SD). At wind speeds 1 SD above average (5.0 + 2.4 = 7.4 m/s), the distance to the WEA increases in average by -38.3 + 64.3 = 26 m when rotor speed increases by 5.2.

```
## [1]  0.0 13.7
```

```
## [1]  0.0 16.8
```

Figure 2.1: Fig 7.

# 3 Appendix 1: How individual random effects account for spatial- and temporal correlation when average effects are of interest (answer to a reviewer comment)

There are more modern statistical methods to analyse movement data such as integrated step selection functions that model the animal movement and habitat selection in the same model, e.g. Thurfjell, Ciuti, and Boyce (2014), Avgar et al. (2016), or Signer et al. (2024). In our analyses, such models would improve the individual-specific estimates of habitat preferences. However, we are primarily interested in the average habitat preferences, i.e. the population-specific habitat preferences. For the population effects, the number of individuals is the crucial sample size, not the number of observations within individuals. Our model contains an individual random effect for every habitat preference (random slope model). This random structure makes that the model takes the number of individuals as the sample size (almost) independent of how many observations there are per individual. Below we demonstrate that principle on a much simplified example.
We use a set of 34 individuals of which we have 10 observation each. Then we replicate the data 10000 times so that of each observation we have 10000 copies (extreme pseudoreplication). When using individual as a random effect the standard error of the resulting estimated intercept does not differ between the original data (10 observations per individual) and the pseudoreplicated (10\*10000 observations per individual) one because the number of individuals does not differ between the two data sets.

```
nind <- 34         # number of individuals
npind1 <- 10       # number of observations per individual in the small data

dat1 <- expand.grid(ind = 1:nind,
                    replication=1:npind1)
set.seed(23523)
indeff <- rnorm(nind)
reseff <- rnorm(npind1)
dat1$y <- indeff[match(dat1$ind, 1:34)] + reseff[match(dat1$replication, 1:npind1)]
dat2 <- data.frame(ind=rep(dat1$ind, 10000),
                   y=rep(dat1$y, 10000)) # replicate the data set 10000 times, 
# i.e. of every observation, we have 10000 pseudoreplicate

mod1 <- lmer(y~1+(1|ind), data=dat1)
mod2 <- lmer(y~1+(1|ind), data=dat2)

summary(mod1)$coefficients
```

```
##              Estimate Std. Error  t value
## (Intercept) 0.4369775   0.158364 2.759324
```

```
summary(mod2)$coefficients
```

```
##              Estimate Std. Error t value
## (Intercept) 0.4369771  0.1583096 2.76027
```

Therefore, the reported population-specific effects in our study do not suffer from pseudoreplication even though our data show, within individual, temporal and spatial correlation. We do not report the individual-specific effect sizes.

# 4 Appendix 2: Merging draws from posterior distributions of different model fits to include uncertainties assessed by a sensitivity analysis (answer to a reviewer comment)

To assess the sensitivity of the results to the randomness in the assignment of the absence points to presence points (pairing of absence with presence points), we created 10 different data sets, each representing a different random assignment of absence points to presence points . We fitted the model to all 10 data sets and show the 10 different results in the effect plots (10 different 95% credible intervals). However, instead of showing 10 different regression lines, we show an average of all 10 regression lines. Similarly, instead of showing 10 values for each parameter estimate in the table, we averaged over the 10 results. To do so, we merged the 4000 draws from the posterior distributions of each of the 10 models resulting in 40000 values from which we reported the mean and the standard deviation as an estimate and a standard error of the parameter. By doing so, the mean corresponds to the average of the 10 different means, whereas the standard deviation is larger than the average standard deviation of the 10 posterior distributions because the standard deviation of the merged posterior distributions also includes the variance that is due to the sensitivity towards the random assignment of the absence points.
A reviewer critised that such a procedure is not correct because the 10 different data sets are not independent. However, we think that for measuring the sensitivity towards a data treatment (here pairing of presence with absence points) the data must not be changed except for the treatment (i.e. here the selection of absence points). Therefore, we think that our approach is valid because it honestly shows the variance that is due to random selection of absence points.

# Referenzen

Avgar, Tal, Jonathan R. Potts, Mark A. Lewis, and Mark S. Boyce. 2016. “Integrated Step Selection Analysis: Bridging the Gap Between Resource Selection and Animal Movement.” *Methods in Ecology and Evolution* 7 (5): 619–30. https://doi.org/10.1111/2041-210X.12528.

Signer, J., J. Fieberg, B. Reineking, U. Schlägel, B. Smith, N. Balkenhol, and T. Avgar. 2024. “Simulating Animal Space Use from Fitted Integrated Step-Selection Functions (iSSF).” *Methods in Ecology and Evolution* 15 (1): 43–50. https://doi.org/10.1111/2041-210X.14263.

Thurfjell, Henrik, Simone Ciuti, and Mark S Boyce. 2014. “Applications of Step-Selection Functions in Ecology and Conservation.” *Movement Ecology* 2 (1): 4. https://doi.org/10.1186/2051-3933-2-4.
