# Supplementary material for: Habitat use of Bechstein´s bats (Myotis bechsteinii) within wind parks in forests
Source: PLoS One. 2026 Mar 25;21(3):e0344730. doi: 10.1371/journal.pone.0344730 (PMC13016307; doi:10.1371/journal.pone.0344730)
Supplement: Attachment — Submitted filename: ReviewerComments_Hurst_etal_PLOSONE.docx [file pone.0344730.s005.docx]

In this manuscript, the authors investigate how wind turbines built in forests influence the roosting and foraging behaviour of two Bechstein’s bat maternity colonies in Germany. Using radio telemetry, they determined the locations of day roosts during summer and identified the areas used at night by tagged individuals. These locations were then analysed in relation to several habitat parameters, as well as to distances from roosts and wind turbines, in order to assess the effects of the latter.

Given the increasing pressure to identify suitable sites for wind farms and the resulting trend to place turbines within forests, this study addresses an important and timely question in the conservation ecology of forest-dwelling bats. The telemetry data collected from these maternity colonies near wind turbines provide a rare opportunity to investigate the effects of forest wind farms on both individual behaviour and colony ecology, going beyond the commonly used approach of acoustic monitoring of bat activity. I think the results are quite interesting and the analysis appears to be well executed and robust. However, to improve readability and clarify the presentation of methods and results, the manuscript would benefit from substantial language revision.

I have a few substantive comments, as well as several minor, mostly linguistic points that should be addressed prior to publication. Below, I outline these points by manuscript section, referring to the line numbers of the manuscript. I have highlighted certain passages as examples that, in my view, could benefit from language improvement, and in some cases I offer possible suggestions for revision. These examples and suggestions are intended for guidance and are not intended to be prescriptive or exhaustive. For example, I have not identified every instance where a comma is needed.

**Abstract**

**L. 9:** “are destroyed” sounds a bit too strong in my opinion. Perhaps something like “may be destroyed and altered” or “are disturbed and altered”

**L. 19 – 20:** Make more clear that this refers to the distance between the bats and the turbines when foraging.

**L. 25:** “curtailments” is a bit unclear here. I am not too familiar with wind farm terminology, but is this the used word for something like “temporal operational restrictions of the wind turbines”? Probably needs some explanation, at least in the introduction.

**L. 25 – 26:** I suggest to change “to limit noise disturbances negative effects” to “to limit the negative effects of noise disturbances”.

**Introduction**

**L. 29:** Add comma after “Worldwide”.

**L. 31:** I guess “alternative” is fine, but perhaps “renewable” or “sustainable” would be more precise.

**L. 31:** I suggest to change “While in the beginning…” to “While initially…”.

**L. 32:** Add a comma after “landscapes”. And I suggest to change “in the last years” to “in more recent years”.

**L. 39:** “There´s”; please avoid contractions in formal or scientific writing and adjust this throughout the manuscript (e.g. “it’s” in L. 43, “didn’t” in L. 46, “didn’t” in L. 113, “doesn’t” in L. 152, …).

**L. 42:** Add a comma after “Those studies” and after “acoustic data”.

**L. 44:** “on a bat colony” – unclear. Do you mean something like “on the roosting behaviour of a bat colony”? Or do you mean on a colony rather than a few (random) individuals?

**L. 46 – 48:** This sentence is a bit unclear to me. What exactly is meant by “avoidance behavior” and how can they avoid the turbines further away from the turbines (> 500m)? Does this just mean that they did avoid the turbines while foraging?

**L. 51:** Probably “Central Europe” is more commonly used.

**L. 59:** Change “rages” to “ranges”.

**L. 63:** Change “a” to “an”.

**L. 64:** Since you did not radio track the entire colonies, consider to change to something like “We tracked members of two maternity colonies…”.

**L. 67:** Add a comma after “Furthermore”.

**Methods**

**L. 75 – 76:** I suggest to change “in a height” to “at an altitude”. Same in L. 85.

**L. 85 – 87:** This sentence needs some rephrasing. Consider splitting it into two sentences.

**L. 91:** Consider changing “used trees” to “roosted in trees” or “used trees as roosts…”.

**L. 92 – 93:** This sentence sounds a bit weird. Do you mean something like “Approval of the wind farm required operation with curtailment algorithms…”. Furthermore, please explain more detailed what curtailment algorithms are and perhaps what the algorithm is particularly for this (and also the other) wind farm.

**L. 100:** Capital letters for “June” and “July”.

**L. 103:** Since you obtained permission to mist-net and use transponders, I assume that is acceptable. However, I was wondering about radio-tracking lactating females during the lactation period. Is this a common practice in your area?

**L. 104:** Change “a operating time” to “an operating time”.

**L. 106:** Change “transmitter” to „transmitters”.

**L. 110:** This depends on the specific journal requirements, but consider spelling out numbers below 10 (e.g., 2 = two), except for before a unit (e.g., 4 km).

**L. 119:** Consider changing “partially” to “sometimes”.

**L. 132:** Add a comma after “HR”. There are several places in the text where additional commas would improve clarity and flow. You might find it helpful to use a grammar tool to check comma placement and ensure readability.

**L. 132:** Change “point” to “points”.

**L. 133:** Change “diameter of breast height” to “diameter at breast height”.

**L. 134:** Replace “on both sides until a distance of 5m” with “within a distance of 5 m on both sides”. And then I would put the “and determined species and DBH” into a separate sentence, e.g. “For each of these trees, we identified the species and determined the DBH.”.

**L. 134 – 135:** Unclear; distance between the trees and what? The transect line?

**L. 136:** Consider replacing “around the point” with “around each point” and add a comma after “point”.

**L. 139:** Here, you could add some units: wind speed (m/s ?), temperature (°C), rotor rotations (rpm). And probably “provided” here fits better than “submitted”.

Furthermore, I wonder whether the fact that the wind turbines have different rotor diameters has an effect on the actual rotational speed, and therefore to what extent the *rpm* of the different wind turbines are comparable. This is probably negligible, but it would be interesting to know if there are any data or literature on noise levels of wind turbines with different diameters or heights.

**L. 148:** Can you elaborate a bit on why you excluded the fixes outside the forest?

**L. 150:** Consider replacing “… resp. the individual” with something like “... or of the respective individual”.

**L. 153:** I suggest to use commas or dashes here around “e.g. between forest and open land”.

**L. 154:** Unclear: how are these strong boundaries taken into account?

**L. 159:** replace “resp. a” with “or”.

**L. 162 – 165:** How many pseudo-absence points were chosen per individual? As many as there were presence points available? Please add this information.

**L. 171:** “data sets” instead of “files”? Check also L. 172.

**L. 172:** Add comma after “Furthermore”.

**L. 174:** I suggest to replace “supposed” with “expected”.

**L. 175:** Which correlation coefficient did you use and how did you define a “strong correlation”? Perhaps you can add a reference where the strength of correlations are defined depending on the coefficient. Of the three cases you mention in lines 176 – 178, two have r < 0.5, which I would not necessarily consider a strong correlation.

**L. 176:** How did you define which variable had the higher ecological meaning? For the three cases you mention in the following sentence, you could explain why you picked which in either case.

**L. 181:** With “actual day roost”, do you mean “current day roost”?

**L. 184 – 185:** I suggest to replace everything after “choice” to “, we included the interaction between distance to the next turbine and all other habitat variable”.

**L. 188:** Add a full stop after “methods”.

**L. 192:** “final model” instead of “end model” and “joined” instead of “united”?

**L. 196 – 197:** How did you assess the autocorrelation in the residuals?

**L. 199 – 200:** It appears that there is something wrong with this sentence. In addition, I would try to write consistently in active voice, like you do in the sentence before, e.g. “For each radio fix, we appended the wind and rotational speed data recorded at the nearest wind turbine at the corresponding time.”

**L. 200 – 202:** I suggest to separate this sentence into two sentences: “If for a radio fix the data of the closest wind turbine was not available, we excluded it from this analysis. This was the case for…”

**L. 202 – 204:** Preferably use active voice: “In a linear model, we used distance to the turbines as response variable and … as explanatory variables. To account for repeated measurements of the same individuals, we added individual ID as random factor.”

Also: adding a random factor probably makes it a generalized linear mixed model, right?

**L. 204 – 205:** “… assessed by visually inspecting the residual distribution”? And how and when was this deemed good enough?

**Results**

**L. 211 (and throughout the results):** Does the value after “±” always refer to the standard deviation? Please add this information somewhere.

**L. 211:** Add “m” after “150”.

**L. 213:** You say they have not moved away further from the wind turbines. It would be neat if you could provide some kind of average distance for periods.

**L. 221:** Add a comma after “PL”. I also suggest adding that the total number of roosts stated here, were during the survey periods and not during the whole summer. Or am I wrong?

**L. 222 – 223 (and throughout the manuscript):** For consistency, you probably want to use points as decimal separator (e.g., 57.2 ha) and commas as thousands separator (e.g., 2,020 m).

**L. 224:** “70 m and” instead of “70 und”.

**L. 224 – 225:** I suggest to replace “all over the wind park” with “across the entire area of the wind farm”.

**L. 226:** Add commas for readability: “…, where roosts had also been found in 2012, …”.

**L. 236 – 238:** I suggest to split this sentence into two sentences.

**L. 238 – 240:** Also consider to split this sentence into two.

**L. 249:** Are you referring here to the maximum distance measured between the recording location and the roost? The word “covered” suggests the actual flight path, which was likely longer than 3.74 km since it was not a direct route, was it?

**L. 268 – 269:** I find it difficult to extract this information from the panel in Figure 7. To me, it appears that as the distance to the wind park increases, the preference for “distance to roost” also increases. Could you clarify what exactly “distance to roost” represents in the figure – does it indicate a smaller or a larger distance?

**L. 269:** “Fig. 7”: It would be helpful if the figure subpanels were labelled (e.g., with letters), so the reader can easily identify which part of the figure is being referenced.

**L. 275 – 278:** I guess it is rather obvious, but also add the information on the meaning of the black line in the figure caption. And is the white line actually white? It appears more greyish to me.

**L. 279 – 286:** Perhaps you want to include this paragraph under its own subheading (e.g. “Influence of wind and rotor rotational speed”).

**L. 279:** “measured at nacelle height”

**L. 280:** Replace “resp.” with “and”.

**L. 284 – 286:** It would be helpful to clarify the distinction between turbines running/operating and rotors merely rotating, and why this distinction matters. Is the difference primarily related to noise?

Also replace “od” with “of” and “while” with “, “

**L. 287 – 290:** Replace “resp.” with “and”.

**Discussion**

**L. 293 – 295:** Given your note in lines 304–305 that the tagged individuals may not have belonged to the same colony, is there any evidence that the individuals found further south in HR in 2007 belonged to the same colony, beyond being the same species and occurring in nearby locations?

**L. 295:** I suggest to start the sentence with something like: “However, despite the turbines being operated for more than 15 years, ...”.

**L. 297:** Replace “possible” with “likely” or “probable”.

**L. 299:** Add commas after “PL” and “turbines”.

**L. 301 – 302:** Consider to change to “The size of the roosting areas was different in both study areas and years, varying between 19 and 105 ha.”.

**L. 304 -305:** “since we didn´t find changes of roosts between all groups of roost trees” – Unclear: do you mean that not all tagged bats always shared the same roosts?

**L. 307:** Replace “those” with “which”.

**L. 312:** Further clarification would be beneficial for the general reader: how does juvenile age influence the duration of consecutive days a roost is occupied?

**L. 313:** “simultaneously” – do you mean during the emergence counts on one evening?

**L. 315:** Please provide a reference for the statement that 'colony sizes of Bechstein’s bats are documented between 10 and 50.' To my knowledge, colony size typically refers to adults only, so a total of over 80 individuals including juveniles may not be unusual. Nevertheless, I agree that this is toward the upper end of the typical colony size for Bechstein’s bats.

**L. 317 – 318:** Since it is not known whether the colony size fluctuated in between these two years, I would rather write something like “Although the colony changed its roosting area over the 15 years during which the wind turbines were in operation, colony size was similar in the two surveyed years.”.

**L. 319:**  “… there were 17 bats counted, of which only one individual was tracked. We presume that these 17 bats were only a subgroup of the entire colony.”

**L. 324:** “patterns” instead of “pattern”

**L. 327:** Do the “foraging search areas” refer to the individual foraging search areas here?

**L. 328:** Consider changing “small” to “relatively small”.

**L. 330:** Replace “at a small space” with “in a small area”.

**L. 335 – 336:** Perhaps more precise: “One individual was recorded foraging at a distance of 4 km to its day roost, …”.

**L. 340 – 341:** I suggest a more concise version: “Furthermore, in areas closer to the turbines, bats showed a preference for habitats characterized by larger tree DBH and reduced herb and shrub layer coverage.”.

**L. 347 – 349:** Unclear, please rephrase.

**L. 356 – 358:** I suggest to rephrase and/or split this sentence into two sentences and try to avoid using “difference” and “different” too often.

**L. 358:** Referring to an earlier comment: it would be useful for the reader if you provided details on the “curtailment regimes” of both wind parks, so it is possible to assess why the one is stricter than the other.

**L. 360 – 361:** delete “a” before “positive” and “the before “habitats”.

**L. 376:** Despite the mild effects for Bechstein’s bats, I think it would be important to note here that wind turbines in forests may exert more pronounced negative impacts on other bat species, for example noctules, which are known to roost in forests but generally forage at higher altitudes. I appreciate that you highlight in lines 391 – 393 that the extent to which your results can be generalized to other bat species is limited, and that further research is needed.

**L. 408:** “forests” instead of “froest”.

**Figures**

**Figures 2, 3, 5 and 6:** I very much like the maps and hope they will be displayed/printed in higher quality in the published version of the manuscript.

**Figure 7:** As I have remarked earlier, consider adding numbers or letters to the subplots for easier referencing (depending on journal requirements).

**Figure 8:** To increase the visibility of the lines, I suggest to display the raw data points smaller and perhaps in fainter colour.
