# Supplementary material for: Habitat use of Bechstein´s bats (Myotis bechsteinii) within wind parks in forests
Source: PLoS One. 2026 Mar 25;21(3):e0344730. doi: 10.1371/journal.pone.0344730 (PMC13016307; doi:10.1371/journal.pone.0344730)
Supplement: Attachment — Submitted filename: Response to reviewers.docx [file pone.0344730.s008.docx]

Dear Lyi Mingyang,

We are pleased to get the great opportunity to publish our results in Plos One! We thank you and each of the three reviewers for the constructive comments and address each point separately (see below, printed in black, for better readability we coloured your and the reviewers remarks in green). We also sent our manuscript to an English native speaking biologist for proof-reading to comply with the reviewers comments on the quality of our English. We have left also the English correction visible with track changes.

The larger changes we conducted as a response to the Reviewer´s comments are addressed with line numbers, which correspond to the “track changes” manuscript version, so they can be easier found.

Unfortunately, the changes in cited literature are not visible, as we use End Note to add literature to the manuscript and this does not support track changes. We added the following references during the revision process: 3., 4., 8., 20., 24., 41., 43.

It was advised, that we should adjust the Competing Interest and the Funding sections. Here are our corrected sections:

Competing interest:

Two of the authors of the study (J.H, R.B.) work for the Freiburg Institute of Applied Animal Ecology, which is a consultancy agency, that also conducts wind farm impact assessment studies which could result in a conflict of interests. Wind farm operators provided the turbine data for the data analysis. However the wind farm operators had no influence at all on study design, data collection, data analysis, interpretation and publication writing and editing. The authors take full responsibility for the integrity of the study and declare, that this does not alter their adherence to  PLOS ONE policies on sharing data and materials.”

Funding:

Two of the authors (J.H., R.B.) are employees of the Freiburg Institute of Applied Animal Ecology, a consultancy agency. The agency got funding for the study by the Federal Agency for Nature Conservation in Germany by means of the Federal Ministery for the Environment, Climate Action, Nature Conservation and Nuclear Safety (FKZ: 3515 86 1000). Members of both institutions took part in regular meetings of a project working group (PAG), where study design and results were discussed. Frauke Krüger from the Federal Agency for Nature Conservation reviewed the manuscript draft.

We hope our revised manuscript now merits a publication in PlosOne.

Best regards,

Johanna Hurst

PONE-D-25-42548

Habitat use of Bechstein´s bats (Myotis bechsteinii) in wind parks in forests

PLOS ONE

Dear Dr. Hurst,

Thank you for submitting your manuscript to PLOS ONE. After careful consideration, we feel that it has merit but does not fully meet PLOS ONE’s publication criteria as it currently stands. Therefore, we invite you to submit a revised version of the manuscript that addresses the points raised during the review process.

Please submit your revised manuscript by Oct 29 2025 11:59PM. If you will need more time than this to complete your revisions, please reply to this message or contact the journal office at [plosone@plos.org](mailto:plosone@plos.org). When you're ready to submit your revision, log on to https://www.editorialmanager.com/pone/ and select the 'Submissions Needing Revision' folder to locate your manuscript file.

Please include the following items when submitting your revised manuscript:

- A rebuttal letter that responds to each point raised by the academic editor and reviewer(s). You should upload this letter as a separate file labeled 'Response to Reviewers'.
- A marked-up copy of your manuscript that highlights changes made to the original version. You should upload this as a separate file labeled 'Revised Manuscript with Track Changes'.
- An unmarked version of your revised paper without tracked changes. You should upload this as a separate file labeled 'Manuscript'.

If you would like to make changes to your financial disclosure, please include your updated statement in your cover letter. Guidelines for resubmitting your figure files are available below the reviewer comments at the end of this letter.

If applicable, we recommend that you deposit your laboratory protocols in protocols.io to enhance the reproducibility of your results. Protocols.io assigns your protocol its own identifier (DOI) so that it can be cited independently in the future. For instructions see: [https://journals.plos.org/plosone/s/submission-guidelines#loc-laboratory-protocols](https://track.editorialmanager.com/CL0/https:%2F%2Fjournals.plos.org%2Fplosone%2Fs%2Fsubmission-guidelines%23loc-laboratory-protocols/1/010f01994b45485c-33f78457-c4b3-4f36-9017-d558c2e7df37-000000/Idl4XTqvlBGXF5NddR8IDFnIDpI_ddw28gBAAzzFs9I=228). Additionally, PLOS ONE offers an option for publishing peer-reviewed Lab Protocol articles, which describe protocols hosted on protocols.io. Read more information on sharing protocols at [https://plos.org/protocols?utm_medium=editorial-email&utm_source=authorletters&utm_campaign=protocols](https://track.editorialmanager.com/CL0/https:%2F%2Fplos.org%2Fprotocols%3Futm_medium=editorial-email%26utm_source=authorletters%26utm_campaign=protocols/1/010f01994b45485c-33f78457-c4b3-4f36-9017-d558c2e7df37-000000/_XmePjoWz2A2M_bmdpVV4ChiqjmqejhhmjJmJ817id4=228).

We look forward to receiving your revised manuscript.

Kind regards,

Lyi Mingyang, Ph.D.
Academic Editor
PLOS ONE

Journal Requirements:

When submitting your revision, we need you to address these additional requirements.

1. Please ensure that your manuscript meets PLOS ONE's style requirements, including those for file naming. The PLOS ONE style templates can be found at

https://journals.plos.org/plosone/s/file?id=wjVg/PLOSOne_formatting_sample_main_body.pdf and

[https://journals.plos.org/plosone/s/file?id=ba62/PLOSOne_formatting_sample_title_authors_affiliations.pdf](https://track.editorialmanager.com/CL0/https:%2F%2Fjournals.plos.org%2Fplosone%2Fs%2Ffile%3Fid=ba62%2FPLOSOne_formatting_sample_title_authors_affiliations.pdf/1/010f01994b45485c-33f78457-c4b3-4f36-9017-d558c2e7df37-000000/v45jaGx2hy1CQPo9XHPIYIezgP9r9Y7qRe1N8ItKJJY=228).

Style was adjusted

2. Thank you for stating the following in the Competing Interests section:

“Two of the authors (J.H, R.B.) work for the Freiburg Institute of Applied Animal Ecology, which is a consultancy agency, that also conducts wind farm impact
assessment studies. Wind farm operators provided the turbine data for the data analysis. However the wind farm operators had no influence at all on study design, data collection, data analysis, interpretation and publication writing and editing. The authors take full responsibility for the integrity of the study.”

We note that one or more of the authors are employed by a commercial company: Freiburg Institute of Applied Animal Ecology

a.        Please provide an amended Funding Statement declaring this commercial affiliation, as well as a statement regarding the Role of Funders in your study. If the funding organization did not play a role in the study design, data collection and analysis, decision to publish, or preparation of the manuscript and only provided financial support in the form of authors' salaries and/or research materials, please review your statements relating to the author contributions, and ensure you have specifically and accurately indicated the role(s) that these authors had in your study. You can update author roles in the Author Contributions section of the online submission form.

Please also include the following statement within your amended Funding Statement.

“The funder provided support in the form of salaries for authors [insert relevant initials], but did not have any additional role in the study design, data collection and analysis, decision to publish, or preparation of the manuscript. The specific roles of these authors are articulated in the ‘author contributions’ section.”

If your commercial affiliation did play a role in your study, please state and explain this role within your updated Funding Statement.

b. Please also provide an updated Competing Interests Statement declaring this commercial affiliation along with any other relevant declarations relating to employment, consultancy, patents, products in development, or marketed products, etc.

Within your Competing Interests Statement, please confirm that this commercial affiliation does not alter your adherence to all PLOS ONE policies on sharing data and materials by including the following statement: "This does not alter our adherence to  PLOS ONE policies on sharing data and materials.” (as detailed online in our guide for authors http://journals.plos.org/plosone/s/competing-interests) . If this adherence statement is not accurate and  there are restrictions on sharing of data and/or materials, please state these. Please note that we cannot proceed with consideration of your article until this information has been declared.

Please include both an updated Funding Statement and Competing Interests Statement in your cover letter. We will change the online submission form on your behalf.

Our cover letter above contains updated versions of Funding and Competing Interests Statements. We are convinced that our affiliations and the role of the funder is explained well now. Please tell us, if we should change anything else.

3. We note that Figures 1-3 & 5-6 in your submission contain [map/satellite] images which may be copyrighted. All PLOS content is published under the Creative Commons Attribution License (CC BY 4.0), which means that the manuscript, images, and Supporting Information files will be freely available online, and any third party is permitted to access, download, copy, distribute, and use these materials in any way, even commercially, with proper attribution. For these reasons, we cannot publish previously copyrighted maps or satellite images created using proprietary data, such as Google software (Google Maps, Street View, and Earth). For more information, see our copyright guidelines: http://journals.plos.org/plosone/s/licenses-and-copyright.

We require you to either (1) present written permission from the copyright holder to publish these figures specifically under the CC BY 4.0 license, or (2) remove the figures from your submission:

a. You may seek permission from the original copyright holder of Figures 1-3 & 5-6 to publish the content specifically under the CC BY 4.0 license.

We recommend that you contact the original copyright holder with the Content Permission Form (http://journals.plos.org/plosone/s/file?id=7c09/content-permission-form.pdf) and the following text:

“I request permission for the open-access journal PLOS ONE to publish XXX under the Creative Commons Attribution License (CCAL) CC BY 4.0 (http://creativecommons.org/licenses/by/4.0/). Please be aware that this license allows unrestricted use and distribution, even commercially, by third parties. Please reply and provide explicit written permission to publish XXX under a CC BY license and complete the attached form.”

Please upload the completed Content Permission Form or other proof of granted permissions as an "Other" file with your submission.

In the figure caption of the copyrighted figure, please include the following text: “Reprinted from [ref] under a CC BY license, with permission from [name of publisher], original copyright [original copyright year].”

 b. If you are unable to obtain permission from the original copyright holder to publish these figures under the CC BY 4.0 license or if the copyright holder’s requirements are incompatible with the CC BY 4.0 license, please either i) remove the figure or ii) supply a replacement figure that complies with the CC BY 4.0 license. Please check copyright information on all replacement figures and update the figure caption with source information. If applicable, please specify in the figure caption text when a figure is similar but not identical to the original image and is therefore for illustrative purposes only.

The following resources for replacing copyrighted map figures may be helpful:

USGS National Map Viewer (public domain): http://viewer.nationalmap.gov/viewer/

The Gateway to Astronaut Photography of Earth (public domain): http://eol.jsc.nasa.gov/sseop/clickmap/

Maps at the CIA (public domain): https://www.cia.gov/library/publications/the-world-factbook/index.html and https://www.cia.gov/library/publications/cia-maps-publications/index.html

NASA Earth Observatory (public domain): http://earthobservatory.nasa.gov/

Landsat: http://landsat.visibleearth.nasa.gov/

USGS EROS (Earth Resources Observatory and Science (EROS) Center) (public domain): http://eros.usgs.gov/#
Natural Earth (public domain): <http://www.naturalearthdata.com/>

For Figure 1 we used Data from the website <http://www.naturalearthdata.com/>, which is not under copyright, like it´s said above. So we confirm, this figure can be printed as it is.

For Figures 2 to 5 we used corine land cover data, which can be downloaded freely from the German BKG (<https://gdz.bkg.bund.de/index.php/default/digitale-geodaten/digitale-landschaftsmodelle/corine-land-cover-5-ha-stand-2018-clc5-2018.html>) and are not copyrighted. The data can be used according to the „data licence Germany – attribution – version 2.0“ (See conditions here: <https://www.govdata.de/dl-de/by-2-0>). We changed the figure captions according to this licence. Nevertheless, as those maps are essential for the publication and we didn´t want to risk problems we asked for the official permission, which is uploaded as „other file“.

4. If the reviewer comments include a recommendation to cite specific previously published works, please review and evaluate these publications to determine whether they are relevant and should be cited. There is no requirement to cite these works unless the editor has indicated otherwise. 

Thanks for that hint, we checked recommendations and cited only the relevant work.

[Note: HTML markup is below. Please do not edit.]

Reviewers' comments:

Reviewer's Responses to Questions

**Comments to the Author**

1. Is the manuscript technically sound, and do the data support the conclusions?

The manuscript must describe a technically sound piece of scientific research with data that supports the conclusions. Experiments must have been conducted rigorously, with appropriate controls, replication, and sample sizes. The conclusions must be drawn appropriately based on the data presented.

Reviewer #1: Yes

Reviewer #2: Yes

Reviewer #3: Yes

2. Has the statistical analysis been performed appropriately and rigorously?

Reviewer #1: Yes

Reviewer #2: Yes

Reviewer #3: Yes

3. Have the authors made all data underlying the findings in their manuscript fully available?

The [PLOS Data policy](http://track.editorialmanager.com/CL0/http:%2F%2Fwww.plosone.org%2Fstatic%2Fpolicies.action%23sharing/1/010f01994b45485c-33f78457-c4b3-4f36-9017-d558c2e7df37-000000/v7nhsV_6L1JD6XtJNN2Hn37x44nHRGXBjoI8LVILWi8=228) requires authors to make all data underlying the findings described in their manuscript fully available without restriction, with rare exception (please refer to the Data Availability Statement in the manuscript PDF file). The data should be provided as part of the manuscript or its supporting information, or deposited to a public repository. For example, in addition to summary statistics, the data points behind means, medians and variance measures should be available. If there are restrictions on publicly sharing data—e.g. participant privacy or use of data from a third party—those must be specified.

Reviewer #1: Yes

Reviewer #2: Yes

Reviewer #3: Yes

4. Is the manuscript presented in an intelligible fashion and written in standard English?

PLOS ONE does not copyedit accepted manuscripts, so the language in submitted articles must be clear, correct, and unambiguous. Any typographical or grammatical errors should be corrected at revision, so please note any specific errors here.

Reviewer #1: Yes

Reviewer #2: No

Reviewer #3: Yes

5. Review Comments to the Author

Please use the space provided to explain your answers to the questions above. You may also include additional comments for the author, including concerns about dual publication, research ethics, or publication ethics. (Please upload your review as an attachment if it exceeds 20,000 characters)

Reviewer #1: The manuscript presents VHF radiotelemetry data from 31 female Bechstein’s bats across two forest wind-parks and uses LoCoH home ranges + conditional logistic models to test whether bats avoid turbine proximity and whether turbine operation (rotor speed / wind) affects distance-to-turbine. It is an interesting, policy-relevant study; however important methodological details, statistical choices, and interpretation require clarification and, in some cases, re-analysis to support the claims.

Introduction
Lines 28 – 36:
To add a short paragraph explicitly distinguishing mortality (strike risk) vs avoidance/behavioral disturbance as separate impacts, and why avoidance is ecologically important (habitat loss, functional landscape fragmentation). This helps readers understand the link from acoustic activity declines to colony-level population effects.

We added a paragraph on this topic (lines 46 to 59)

Lines 33 – 34:
I think that you should add these two important references as examples to support your sentence: “Wind turbines can have severe negative impacts on wildlife”. I would like to suggest:
Smeraldo, S., Bosso, L., Fraissinet, M., Bordignon, L., Brunelli, M., Ancillotto, L., & Russo, D. (2020). Modelling risks posed by wind turbines and power lines to soaring birds: The black stork (Ciconia nigra) in Italy as a case study. Biodiversity and Conservation, 29(6), 1959-1976.
Estellés‐Domingo, I., & López‐López, P. (2025). Effects of wind farms on raptors: A systematic review of the current knowledge and the potential solutions to mitigate negative impacts. Animal Conservation, 28(3), 334-352.

We added the proposed reference from Estellés‐Domingo, I., & López‐López, P. (2025) and a further reference on general impacts of wind power to the environment. We didn´t add the reference on the black stork as it concentrates on one species and is therefore not really fitting to underline the general problem

Lines 41 – 47:
To discuss briefly the spatial and sampling biases inherent to acoustics (detector detection radius, weather dependence) and that some acoustic studies used paired automatic recorders at multiple distances (give example refs) — this makes the contrast to telemetry more explicit.

Acoustic limitations are discussed now (lines 63 to 68)

Lines 49 – 69:
To add an explicit statement of hypotheses (e.g., H1: colonies avoid areas near operating turbines; H2: avoidance increases with rotor rotation/wind speed; H3: high-quality habitat will mitigate avoidance) — this will sharpen the later tests.

Hypotheses are stated now (lines 102-106)

**Materials & Methods**
Lines 72 – 94:
Good to include pre-construction colony knowledge — strengthens inference about persistence. But the temporal mismatch (HR turbines installed ~2004–2006; PL installed 2019) creates heterogeneity in exposure history. Authors should explicitly describe how they account for differences in turbine age and earlier pre-construction baseline surveys when interpreting long-term effects.
The description of curtailment is brief — authors must fully report curtailment thresholds and implementation periods (dates/hours) because curtailment both affects collision risk and could change noise/operation patterns that the study tests. If operators provided curtailment rules, report exact thresholds and any temporal changes. If not available, state that and discuss implications.

It is true, that the temporal mismatch between the two study sites is not ideal for the data analysis. The sample size for a seperate analysis of both areas was too low to get reliable results. When we analyse the data together we have to assume, that the animals show the same reactions regarding their habitat preferences. We are convinced that this assumption is true, as despite of the temporal mismatch, in both areas the colonies were still present, with similar numbers of individuals and in close distances to the turbines. Also, the habitat parameters are similar in the both areas, so a similar reaction oft he bats can be expected.

We added some information to the curtailments as this was also asked by the other reviewers (lines 143-148). We cannot add the explicit algorithms, as that is not allowed by the operators. But it is now clarified, that the algorithms in PL are higher than in HR also influencing the noise patterns.

Lines 92 – 122:
I think that 31 individuals is reasonable for VHF work on a small forest bat. But each bat tracked only 2–4 nights (short individual monitoring periods). This limits inference about within-individual behavioral variability and temporal response to turbine operation. Compare: many VHF studies of forest bats track individuals for longer periods (e.g., week(s) of continuous tracking) or complement with acoustic sampling (see references cited in manuscript). Recommend authors justify why 2–4 nights gives an unbiased sample of foraging choices (e.g., argument about territoriality and repeated use of core patches).
Cross-bearing VHF is subject to angular error — excluding angles <30° / >150° is standard, but authors should quantify expected locational error (e.g., median error from calibration tests or from simultaneous ground-truthing). If no calibration, estimate error using geometry and observer positions; report a confidence radius for each fix or a mean error. Many telemetry studies report mean triangulation error (e.g., 20–100 m depending on forest). Not accounting for variable locational error can bias LoCoH area estimation and distance-to-turbine measures.
Turbine data were provided at 10-minute resolution. Bearing intervals were 3 min. Authors should explain how they matched fix timestamps to the 10-min turbine dataset (nearest 10-min window? interpolation?). Precise matching procedure affects the wind/rotor analysis.
Authors reduced autocorrelation by using every third fix in the RSF-style analysis — this is pragmatic but coarse. Alternative modern approaches (see below) can model movement and selection jointly (iSSF) or use mixed-effects RSF with correlated random effects. Compare with integrated step selection functions (Thurfjell et al., Avgar et al.) which explicitly account for movement autocorrelation and availability along steps; this is more appropriate for fine temporal telemetry data. Recommend either: (i) re-analyse using iSSF (if step data possible) or (ii) quantify residual autocorrelation after subsampling (e.g., variogram), and show sensitivity to subsampling choice.

We justified the short tracking time in the methods (lines 168,169). We are convinced that this is appropriate and it produces better data to track more individuals than extend tracking time per individual.

Bearing errors are really difficult to measure. We tried to reduce them as good as possible by using positions for the bearings as close to the animals as possible, so we could be sure in most of the times, that the animal was foraging in the adjacent forest area. Furthermore, bearing errors are not systematic, but random errors that produce a noise in the data. Consequently, bearing errors do not introduce a bias but only increase variance. By taking a large number of fixes, which all have a small random error, we nevertheless get a good result for the foraging and core areas of the animals.

The turbine data is reported in 10-min intervals, but the values for wind speed and rotor rotation are mean values for the last 10 minutes, so we matched every fix to the corresponding 10-min-interval.

We agree that there are more modern statistical methods to analyse movement data than we used. For example, integrated step selection functions that model the animal movement and habitat selection in the same model, e.g. Thurfjell, Ciuti, and Boyce (2014), Avgar et al. (2016), or Signer et al. (2024). In our analyses, such models may improve the individual-specific estimates of habitat preferences, whereas the average habitat preference (averaged over all individuals) may be improved only little. We are primarily interested in the average habitat preferences. For the estimation of population effects, the number of individuals is the crucial sample size, not the number of observations within individuals, thus thinning has a negligible effect on the estimate of the average habitat preference. Our model contains an individual random effect for every habitat preference estimate (random slope model). With this random structure the model takes the number of individuals as the sample size (almost) independent of how many observations there are per individual. Thus, thinning might not have been needed at all because the random individual effect is correcting for that pseudoreplication. We added this information to the methods section.

We also explain this in the new supporting information S2, an html-document, where we also show our R-codes.

Line 131: To all the R codes used in this study in the supplementary materials.

The R code is now added with some remarks in the new S2 html-document and S3 rmd-file.

Lines 123–137:
Mapping design seems thorough. Key point: presence points (fixes) were assigned habitat variables from the next mapping point in the same vegetation unit. This assignment method can introduce misclassification if mapping resolution is coarse relative to fix error. Recommend: (i) explicitly state mean distance from each telemetry fix to the assigned mapping point; (ii) test sensitivity by using the nearest mapping point vs. within-unit average; (iii) include mapping error/account for misclassification in the methods or discussion.
LoCoH is a valid choice to respect sharp habitat boundaries (Getz et al. 2007), and they cite that. Good. However, LoCoH performance can be sensitive to the ‘a’ parameter; choosing a = largest distance between two positions may lead to overly large radii for widely dispersed individuals. Authors should show sensitivity analysis of LoCoH size to ‘a’ parameter (e.g., alternative a values, cross-validation). Alternative methods: kernel density estimation (KDE) with bandwidth selection, biased random bridges (for movement autocorrelation), or AKDE (Autocorrelated KDE; using ctmm) which explicitly accounts for temporal autocorrelation and yields confidence intervals for home-range area. Suggest discussing these alternatives and justifying LoCoH over e.g., AKDE or BRB.

It´s true, that all these recommendations would contribute to make the analysis more fine-scaled. We know, that we have noise in the data through errors in fixes, correlation to mapping data and the choice of parameters in the LoCoH-analysis. Nevertheless, we tried to minimize those errors e.g. by position choice in bearing (see above), a fine-scale habitat mapping, and by choosing commonly used methods in the LoCoH-analysis. The applied methods produced expectable and reasonable results. So the proposed sensitivity analysis is dispensable, as it would not deliver any additional findings.

Lines 149 – 150:
I think that you should add these two important references as examples to support your sentence: “The roosting area and the home range of the colony and the individals was calculated as minimum convex polygon (MCP) of all radio fixes of the colony resp.”. I would like to suggest:
Ancillotto, L., Palmieri, A., Canfora, C., Nastasi, C., Bosso, L., & Russo, D. (2022). Spatial responses of long-eared bats Plecotus auritus to forestry practices: implications for forest management in protected areas. Forest Ecology and Management, 506, 119959.
Kurek, K., Gewartowska, O., Tołkacz, K., Jędrzejewska, B., & Mysłajek, R. W. (2020). Home range size, habitat selection and roost use by the whiskered bat (Myotis mystacinus) in human-dominated montane landscapes. PloS one, 15(10), e0237243.

Thanks fort he hint to add references here. We decided to add the first one that you proposed, and a second on the Bechstein´s bat itself, which was already cited in the manuscript and also uses this method

Lines 159 – 197:
Using conditional logistic regression (clogit) on matched pairs (use/available) is a standard case-control RSF approach. That is fine. However: the choice of pseudo-absence locations drawn from mapping points within 95% LoCoH — while reasonable — should be justified: mapping points are not uniformly distributed and represent sampling locations, not necessarily unbiased availability. Better would be to sample availability uniformly across the 95% LoCoH polygon (with or without habitat-stratified sampling) or to weight pseudo-absences by mapping effort. The current design risks selection bias if mapping density varies.

We are convinced, that the mapping points represent the available habitat well. We put a large effort in the mapping and distributed the mapping points evenly above the home range of the whole colonies with one mapping point and transect per hectare which is fine scaled enough to record the small scale difference in forest structure.

Creating 10 datasets to assess random pairing is ad-hoc. A more statistically coherent approach is to use multiple imputation or to sample a large set of random available points per used fix and fit a hierarchical model that integrates uncertainty (or fit a conditional logistic with many available per used).

All methods listed above could be named “ad-hoc” and we disagree that some of them are “statistically more coherent” (whatever that means?) than others. The problem that all these methods try to solve is that the available points are randomly chosen and thus, if we would repeat the study, we would get different results. Our approach is a valid sensitivity analysis.

Combining posterior draws from separate dataset fits (i.e., uniting the 4,000 draws from each of 10 datasets) is questionable statistically because it treats the datasets as independent replicates when they are not (they are different random draws from the same underlying availability). A hierarchical Bayesian model that treats availability sampling as part of the model (or fits a single model with many available points per used) is preferable. If authors keep the 10-dataset approach, they must justify pooling procedure formally (e.g., show results are stable across datasets and present median estimates and variability across datasets rather than literally merging posteriors).

Combining draws from separate distributions is a common method in Bayesian statistics to take into account uncertainties appearing at different levels of the analysis. For example, for obtaining draws from a posterior predictive distribution, draws are combined from different data distributions each defined by a specific draw of the joint posterior distribution of the model parameters. As a result, the posterior predictive distribution shows how the modelled data distribution looks like taking into account the uncertainty in the parameter estimates (see. e.g. Gelman et al. 2014 Bayesian Data Analysis, CRC Press). In our case, combining the posterior distributions obtained by the 10 different data sets results in a posterior distribution that takes into account the uncertainty that is due to the random selection of available points leading to the 10 different data sets. This procedure does not assume that the 10 data sets are independent, in contrary, it would be wrong to combine draws from posterior distributions in case of 10 independent data sets. In that case, we would rather average over the 10 data sets which would lead to a more precise estimate.

We now explain the procedure more explicitly in supplement S2.
Authors must demonstrate that this subsampling adequately reduces temporal autocorrelation (e.g., show semivariogram or autocorrelation function pre/post subsampling). Many modern studies use mixed models with autocorrelation structures or iSSF to directly model movement and selection. Recommend at least a sensitivity check (every 2nd, 4th fixes) and reporting how estimates change.
removed correlated variables (r>0.8 for leaf/herb). Good, but should present a correlation matrix (Supplement) and Variance Inflation Factors (VIFs) for final variables.

We now present a semi-variogram and a correlation matrix in the supplementary material.

Authors state individual ID was random effect — good. But conditional logistic with random intercepts in stan_clogit must be carefully parameterized; clarify how random effects were implemented (by stratum or as intercepts) and report between-individual variance.

Yes, it is true that specifying random effects in conditional logistic regression is tricky and different from non-conditional models, and it is also true that we did not specify how we did it. We now explain how the random individual effect was implemented and we also define what we used as a stratum (lines 278-283).

They report checking R̂ and effective sample size. Good; include R̂ values and n_eff in Supplement. Also report posterior predictive checks, and ideally model comparison (e.g., WAIC / LOO-CV) for models with/without turbine interaction terms.
They included interactions between distance to turbine and habitat variables. This is important. But interactions can be nonlinear — consider smoothing (GAM) or categorize distance bands (e.g., 0–100 m, 100–300 m, >300 m) to test nonlinearity or check the linearity assumption on logit scale.

We now give the R-hat and n_eff in the supplement S2. We refrain from including a smoothing term as the number of parameters in relation to sample size already is quite high in our models and we are afraid of describing random data structures when using smoothers or categorical distance bands.

Lines 197 – 205:
Distance to turbine is nonnegative, skewed, and bounded by study geometry. Authors must show residual diagnostics (they say visually assessed) and consider transformations (log distance) or GLMM families if residuals deviate. Also spatial nonindependence (fixes clustered in space) may violate assumptions; consider spatial random effects or include spatially structured error (e.g., via INLA/GAM).

We now show the distribution of the residuals in the supplement S2.

The effect of rotor speed/wind on avoidance may be nonlinear with thresholds (e.g., turbines start above a cut-in speed). Authors hint at thresholds (different in HR and PL). Consider testing for breakpoints (piecewise regression) or including turbinerunning (on/off) as a categorical variable. Report the cut-in speeds and rotor RPM thresholds explicitly.
They assigned turbine metrics of the “next turbine” to each fix. Clarify how “next turbine” is defined (closest by Euclidean distance?); if a bat is between turbines, its behavior may respond to multiple turbines simultaneously. Consider aggregating metrics across nearest k turbines (e.g., nearest 3) weighted by inverse distance or modeling turbine presence as a spatial covariate (distance to nearest turbine + whether nearest turbine is running).
Curtailment means turbines may be turned off at low wind speeds (and bat activity may be highest at those low speeds). This confounds wind speed, rotor rotation and bat distance. Authors should present turbine operational state (running vs stopped) as separate predictor and test its effect.

The thresholds for wind speed in the curtailment algorithm vary per month and night time, due to that it´s not possible to introduce a breakpoint into the model.

We are convinced, that the distance to the next turbine, it was defined by Euclidean distance, is the best value to measure disturbance effects as the next turbine is the one wich has strongest emmissions. When we would aggregate several turbines, which sometimes are not very close, that would increase the noise in the data.

Rotor rotation is a proxy for disturbances of the rotor blades, that is more informative than the operational state, which only states if the turbine is running or not. It is not necessary to add this as seperate predictor to the model.

**Results**
Lines 206 – 240:
Manuscript: HR: 14 roost trees (6 in 2019, 9 in 2022); roost distances to turbines mean 415 ± 225 m (range 150–811 m). PL: 26 roost trees; roost-turbine distances mean 295 ± 161 m (range 70–684 m). Colony sizes reported from emergence counts (max 88 in HR, 59 in PL).
Present roost coordinates or a table (S1) with roost IDs, dates, and nearest turbine distance — this helps reproducibility. If privacy concerns exist, provide masked coordinates but with exact distances to turbines.
The narrative on roost movement (HR core moving north compared to 2007) should be supported with statistical comparison (e.g., difference in centroid distance to turbines between years) rather than visual description. Provide metrics with confidence intervals.

We provide now a S1 Table S2 with roost ID, further information on the roost and the exact distance to the turbines. Furthermore, for HR we provide now the metrics on the distance between roost trees and turbines in 2007, so it´s easier to compare the data.

Lines 241 – 259:
Manuscript: Colony MCPs sizes, distribution of fixes relative to roost (percent within 500 m and 1,000 m), LoCoH 95% and 50% ranges, core areas.
To provide tables with individual MCP and LoCoH areas (they mention Table S3 — ensure it is comprehensive). Show variability across individuals and years.
The authors report large variability in LoCoH sizes — include a sensitivity test showing how LoCoH a-parameter choice affects results.
The test for territoriality (core areas overlap) is descriptive; consider quantifying overlap (Bhattacharyya index, utilization distribution overlap index) and test whether overlap is less than expected by chance.

All data is presented in S1 Table S4, it also shows the variability between individuals and years. As the use of maximum distance between two points as „a“ is a commonly used method to calculate the LoCoHs for bat species with small home ranges, we used this in our analysis. We think that a sensitivity analysis would be beyond the aim of this paper, as we don´t want to value the different methods to calculate core foraging area. The same is true fort he overlap oft he core areas, as the territoriality of the Bechstein´s bats is not the main topic of this paper. With too much side analysis the manuscript would loose ist focus.

Lines 267 – 277:
Bats used habitats close to turbines mainly when close to their roosts and when high DBH and low shrub/herb cover (high quality). The distance to turbines increased with rotor rotation at high wind speeds; different thresholds between HR (8 m/s) and PL (12 m/s). Figures present model outputs (Fig 7, Fig 8).
Present the model coefficients (posterior means and 95% CIs) in a table for the final model (they refer to S1 Table S4 — ensure this is included). For interaction terms, present marginal effect plots (they have Fig 7 — good) but also show intervals across individuals, because heterogeneity could be large.
On pooling posterior draws from 10 datasets: instead of pooling, present parameter estimates per dataset to show stability, and then report combined estimates using formal model averaging or hierarchical pooling. The current description (“the 4,000 simulated values from each of the 10 data sets were united and used to describe the averaged effects”) is ambiguous and statistically weak; explain procedure and justify.
For the wind/rotor model: show predicted distances for representative roost distances and study areas; show whether effect sizes are biologically meaningful (e.g., how many meters change per 1 m/s). Report effect magnitudes with credible intervals so readers can judge ecological significance.

We decided to add the coefficient tables to the manuscript instead of the supplementary material, as we agree that this information is crucial to interpret the results. This also enables judgement of affect sizes. An more detailled example is given in the new S2, the html-Document with the r-Script and further remarks. We refrain from showing the effects for each individual separately because we focus on the population mean. However, we now show the among individual variance for each effect in the table.

The topic of the pooling of the results of the 10 datasets is already answered at the remark regarding the same question in the methods.

Discussion
Lines 291 – 321:
Manuscript conclusion: colonies did not abandon roosts near turbines; colony sizes remain high. This is a central and important finding.
Persistence does not equal absence of negative effects. The authors acknowledge this, but must discuss alternative possibilities: demographic compensation (e.g., immigration), delayed population declines, or reduced fitness (lower juvenile survival) not detected by short tracking windows. Recommend tempering the language and calling for long-term demographic monitoring. Also discuss that the colonies may tolerate turbines up to a threshold of turbine density/operation.

Thanks for that good comment, we included those points into the discussion (lines 485-493)

Lines 337 – 350:
Bats prefer high-quality habitat close to roosts despite turbines; further from roosts they avoid turbine-proximate foraging. This aligns with acoustic studies showing activity declines near turbines. Good linkage.
To discuss how the limited tracking nights and possible seasonal effects (they tracked only late June–July) may bias detection of avoidance (e.g., seasonal changes in foraging range/juvenile mobility). Recommend recommending multi-season tracking.

Thanks, we also added this point to the discussion (lines 534-545)

Lines 351–360:
The authors infer that increased rotor rotation (and assumed noise) leads to increased distance; this is plausible and supported by other studies (they cite Ellerbrok et al.).
Rotor rotation may also correlate with other cues (vibration, infrasound, blade movement airflows). To support noise mechanism more directly, authors could (in future) include in-forest sound pressure level (SPL) measurements at different distances and turbine operational states — this would strengthen causal inference. At a minimum, the discussion should explicitly note that rotor RPM is a proxy for noise, not a direct measurement.

We noted that the increasing rotor rotation causes noise emmissions. We were thinking about measuring those, but it´s really difficult to realise that, especially for ultrasonic noise.

**Reviewer #2:**

In this manuscript, the authors investigate how wind turbines built in forests influence the roosting and foraging behaviour of two Bechstein’s bat maternity colonies in Germany. Using radio telemetry, they determined the locations of day roosts during summer and identified the areas used at night by tagged individuals. These locations were then analysed in relation to several habitat parameters, as well as to distances from roosts and wind turbines, in order to assess the effects of the latter.

Given the increasing pressure to identify suitable sites for wind farms and the resulting trend to place turbines within forests, this study addresses an important and timely question in the conservation ecology of forest-dwelling bats. The telemetry data collected from these maternity colonies near wind turbines provide a rare opportunity to investigate the effects of forest wind farms on both individual behaviour and colony ecology, going beyond the commonly used approach of acoustic monitoring of bat activity. I think the results are quite interesting and the analysis appears to be well executed and robust. However, to improve readability and clarify the presentation of methods and results, the manuscript would benefit from substantial language revision.

I have a few substantive comments, as well as several minor, mostly linguistic points that should be addressed prior to publication. Below (in the attached document), I outline these points by manuscript section, referring to the line numbers of the manuscript. I have highlighted certain passages as examples that, in my view, could benefit from language improvement, and in some cases I offer possible suggestions for revision. These examples and suggestions are intended for guidance and are not intended to be prescriptive or exhaustive. For example, I have not identified every instance where a comma is needed.

General comment: Thank you for your detailled comments, also on verbal expressions and commata. We did a language revision of the whole manuscript by an English native speaker. We adopted all your language corrections first, as it´s said in our answers below, but it´s possible that during the correction process some phrases changed again.

**Abstract**

**L. 9:** “are destroyed” sounds a bit too strong in my opinion. Perhaps something like “may be destroyed and altered” or “are disturbed and altered”

Phrase was changed

**L. 19 – 20:** Make more clear that this refers to the distance between the bats and the turbines when foraging.

“Foraging” was added

**L. 25:** “curtailments” is a bit unclear here. I am not too familiar with wind farm terminology, but is this the used word for something like “temporal operational restrictions of the wind turbines”? Probably needs some explanation, at least in the introduction.

The word curtailment was changed here and explained later in the section “Study area”.

**L. 25 – 26:** I suggest to change “to limit noise disturbances negative effects” to “to limit the negative effects of noise disturbances”.

Phrase was changed

**Introduction**

**L. 29:** Add comma after “Worldwide”.

Done

**L. 31:** I guess “alternative” is fine, but perhaps “renewable” or “sustainable” would be more precise.

We use “sustainable” now

**L. 31:** I suggest to change “While in the beginning…” to “While initially…”.

Done

**L. 32:** Add a comma after “landscapes”. And I suggest to change “in the last years” to “in more recent years”.

Done

**L. 39:** “There´s”; please avoid contractions in formal or scientific writing and adjust this throughout the manuscript (e.g. “it’s” in L. 43, “didn’t” in L. 46, “didn’t” in L. 113, “doesn’t” in L. 152, …).

We changed this in the complete manuscript

**L. 42:** Add a comma after “Those studies” and after “acoustic data”.

Sentence changed in revision process

**L. 44:** “on a bat colony” – unclear. Do you mean something like “on the roosting behaviour of a bat colony”? Or do you mean on a colony rather than a few (random) individuals?

We clarified that, also by stressing the problems of acoustic data, which was recommended by another reviewer (lines 63-71).

**L. 46 – 48:** This sentence is a bit unclear to me. What exactly is meant by “avoidance behavior” and how can they avoid the turbines further away from the turbines (> 500m)? Does this just mean that they did avoid the turbines while foraging?

Yes, it means an avoidance while foraging, we added that.

**L. 51:** Probably “Central Europe” is more commonly used.

Changed

**L. 59:** Change “rages” to “ranges”.

Done

**L. 63:** Change “a” to “an”.

Done

**L. 64:** Since you did not radio track the entire colonies, consider to change to something like “We tracked members of two maternity colonies…”.

Changed to individuals of colonies

**L. 67:** Add a comma after “Furthermore”.

Sentence was changed.

**Methods**

**L. 75 – 76:** I suggest to change “in a height” to “at an altitude”. Same in L. 85.

Done

**L. 85 – 87:** This sentence needs some rephrasing. Consider splitting it into two sentences.

Done

**L. 91:** Consider changing “used trees” to “roosted in trees” or “used trees as roosts…”.

Done

**L. 92 – 93:** This sentence sounds a bit weird. Do you mean something like “Approval of the wind farm required operation with curtailment algorithms…”. Furthermore, please explain more detailed what curtailment algorithms are and perhaps what the algorithm is particularly for this (and also the other) wind farm.

The applied curtailment algorithm is explained more detailed (lines 141 to 148)

**L. 100:** Capital letters for “June” and “July”.

Done

**L. 103:** Since you obtained permission to mist-net and use transponders, I assume that is acceptable. However, I was wondering about radio-tracking lactating females during the lactation period. Is this a common practice in your area?

The radiotracking of lactating individuals is common here, it is regularly done to find maternity roosts in forests as there is no other good method for that. The exception are females in their late pregnancy state, they shouldn´t be stressed with more weight.

**L. 104:** Change “a operating time” to “an operating time”.

Done

**L. 106:** Change “transmitter” to „transmitters”.

Done

**L. 110:** This depends on the specific journal requirements, but consider spelling out numbers below 10 (e.g., 2 = two), except for before a unit (e.g., 4 km).

We corrected that throughout the manuscript

**L. 119:** Consider changing “partially” to “sometimes”.

Done

**L. 132:** Add a comma after “HR”. There are several places in the text where additional commas would improve clarity and flow. You might find it helpful to use a grammar tool to check comma placement and ensure readability.

We did a verbal and grammatical revision by a native speaker, we hope it´s correct now.

**L. 132:** Change “point” to “points”.

Done

**L. 133:** Change “diameter of breast height” to “diameter at breast height”.

Done

**L. 134:** Replace “on both sides until a distance of 5m” with “within a distance of 5 m on both sides”. And then I would put the “and determined species and DBH” into a separate sentence, e.g. “For each of these trees, we identified the species and determined the DBH.”

Done

**L. 134 – 135:** Unclear; distance between the trees and what? The transect line?

Yes, we added that.

**L. 136:** Consider replacing “around the point” with “around each point” and add a comma after “point”.

Done

**L. 139:** Here, you could add some units: wind speed (m/s ?), temperature (°C), rotor rotations (rpm). And probably “provided” here fits better than “submitted”.

Furthermore, I wonder whether the fact that the wind turbines have different rotor diameters has an effect on the actual rotational speed, and therefore to what extent the *rpm* of the different wind turbines are comparable. This is probably negligible, but it would be interesting to know if there are any data or literature on noise levels of wind turbines with different diameters or heights.

Noise levels are influenced by different factors, the most important factor is the turbine type (with or without gearbox), but the rotor diameter also plays a role, that´s true. The topic is too wide to be discussed in the manuscript, but we stressed the fact, that noise levels can be different.

**L. 148:** Can you elaborate a bit on why you excluded the fixes outside the forest?

We clarified that in the manuscript (lines 218 -223)

**L. 150:** Consider replacing “… resp. the individual” with something like “... or of the respective individual”.

Done

**L. 153:** I suggest to use commas or dashes here around “e.g. between forest and open land”.

Done

**L. 154:** Unclear: how are these strong boundaries taken into account?

The boundaries can appear as those in the dimensions of the convex hulls as they are not smoothed out. This is easier to understand when you have a look at the cited literature. We think, it´s not necessary to explain it in more detail inside the manuscript.

**L. 159:** replace “resp. a” with “or”.

Done

**L. 162 – 165:** How many pseudo-absence points were chosen per individual? As many as there were presence points available? Please add this information.

As it is said in the manuscript, we used pairs of presence of absence points, so it should be clear that we chose as many absence points as presence points.

**L. 171:** “data sets” instead of “files”? Check also L. 172.

Done

**L. 172:** Add comma after “Furthermore”.

Done

**L. 174:** I suggest to replace “supposed” with “expected”.

Done

**L. 175:** Which correlation coefficient did you use and how did you define a “strong correlation”? Perhaps you can add a reference where the strength of correlations are defined depending on the coefficient. Of the three cases you mention in lines 176 – 178, two have r < 0.5, which I would not necessarily consider a strong correlation.

There is no threshold that can be applied in all situations because what “strong” means depends on many factors. Further, it is not forbidden (it does not introduce any bias or error) to have correlated variables as predictors in a model (see, e.g.: <https://tobiasroth.github.io/BDAEcology/lm.html#collinearity>). When selecting predictor variables, we were considering the correlation coefficients to identify groups of variables that may measure a similar ecological mechanism. Then, we discussed the ecological mechanisms that may lead to a habitat preference and chose those that most closely measured that mechanism.

**L. 176:** How did you define which variable had the higher ecological meaning? For the three cases you mention in the following sentence, you could explain why you picked which in either case.

We added that in the manuscript (lines 261-267)

**L. 181:** With “actual day roost”, do you mean “current day roost”?

Yes, we corrected that.

**L. 184 – 185:** I suggest to replace everything after “choice” to “, we included the interaction between distance to the next turbine and all other habitat variable”.

Done

**L. 188:** Add a full stop after “methods”.

Done

**L. 192:** “final model” instead of “end model” and “joined” instead of “united”?

Done

**L. 196 – 197:** How did you assess the autocorrelation in the residuals?

We used acf-plots for measuring temporal autocorrelation and we used semi-variograms to measure spatial correlation.

**L. 199 – 200:** It appears that there is something wrong with this sentence. In addition, I would try to write consistently in active voice, like you do in the sentence before, e.g. “For each radio fix, we appended the wind and rotational speed data recorded at the nearest wind turbine at the corresponding time.”

Sentence was corrected.

**L. 200 – 202:** I suggest to separate this sentence into two sentences: “If for a radio fix the data of the closest wind turbine was not available, we excluded it from this analysis. This was the case for…”

Sentence was split.

**L. 202 – 204:** Preferably use active voice: “In a linear model, we used distance to the turbines as response variable and … as explanatory variables. To account for repeated measurements of the same individuals, we added individual ID as random factor.”

Also: adding a random factor probably makes it a generalized linear mixed model, right?

Sentence was corrected.

Adding a random factor makes it a linear mixed model instead of a linear model. The “mixed” was missing, we added that.

**L. 204 – 205:** “… assessed by visually inspecting the residual distribution”? And how and when was this deemed good enough?

We give the residual plots in the supplementary material S2 now. What is good enough depends on the context of the study, the purpose and many other factors. Therefore, we let the readers judge themselves how much they trust the model.

**Results**

**L. 211 (and throughout the results):** Does the value after “±” always refer to the standard deviation? Please add this information somewhere.

This information was added at the first occurrence.

**L. 211:** Add “m” after “150”.

Done

**L. 213:** You say they have not moved away further from the wind turbines. It would be neat if you could provide some kind of average distance for periods.

That´s a good idea, we provide the distances for 2007 now in the manuscript (lines 320-322).

**L. 221:** Add a comma after “PL”. I also suggest adding that the total number of roosts stated here, were during the survey periods and not during the whole summer. Or am I wrong?

That´s true, we changed the sentence to “we found 26 roost trees”

**L. 222 – 223 (and throughout the manuscript):** For consistency, you probably want to use points as decimal separator (e.g., 57.2 ha) and commas as thousands separator (e.g., 2,020 m).

Done

**L. 224:** “70 m and” instead of “70 und”.

Done

**L. 224 – 225:** I suggest to replace “all over the wind park” with “across the entire area of the wind farm”.

We replaced the first part; wind park was used throughout the manuscript so we leave it here.

**L. 226:** Add commas for readability: “…, where roosts had also been found in 2012, …”.

Done

**L. 236 – 238:** I suggest to split this sentence into two sentences.

Done

**L. 238 – 240:** Also consider to split this sentence into two.

Done

**L. 249:** Are you referring here to the maximum distance measured between the recording location and the roost? The word “covered” suggests the actual flight path, which was likely longer than 3.74 km since it was not a direct route, was it?

That´s true, we corrected that, referring only to the distance between points.

**L. 268 – 269:** I find it difficult to extract this information from the panel in Figure 7. To me, it appears that as the distance to the wind park increases, the preference for “distance to roost” also increases. Could you clarify what exactly “distance to roost” represents in the figure – does it indicate a smaller or a larger distance?

Preference values of 1 mean that there´s no preference, the according structure is used as offered. Values above 1 indicate a preference, below 1 an avoidance. For the variable distance to roost that means, that when distance to the turbine is small, the Bechstein bat´s prefer small distances to the roosts, further away from the turbines they prefer long distance to the roosts. We added this explanation, that should make it clearer.

**L. 269:** “Fig. 7”: It would be helpful if the figure subpanels were labelled (e.g., with letters), so the reader can easily identify which part of the figure is being referenced.

Latters were added and referenced in the text

**L. 2Fig5 – 278:** I guess it is rather obvious, but also add the information on the meaning of the black line in the figure caption. And is the white line actually white? It appears more greyish to me.

There was a mistake, we described the black line, we corrected that. We also added an explanation for the grey line.

**L. 279 – 286:** Perhaps you want to include this paragraph under its own subheading (e.g. “Influence of wind and rotor rotational speed”).

We left it like it is, as this topic is also linked to the foraging behaviour, another subheading would be misleading.

**L. 279:** “measured at nacelle height”

Done

**L. 280:** Replace “resp.” with “and”.

Done

**L. 284 – 286:** It would be helpful to clarify the distinction between turbines running/operating and rotors merely rotating, and why this distinction matters. Is the difference primarily related to noise?

We clarified that in the manuscript. The difference is caused by different curtailment regimes, the turbines start to operate at different speeds. So noise disturbances started at different wind speeds in the both parks (lines 423-427).

Also replace “od” with “of” and “while” with “, “

Done

**L. 287 – 290:** Replace “resp.” with “and”.

Done

**Discussion**

**L. 293 – 295:** Given your note in lines 304–305 that the tagged individuals may not have belonged to the same colony, is there any evidence that the individuals found further south in HR in 2007 belonged to the same colony, beyond being the same species and occurring in nearby locations?

We can´t know that for sure, but we are quite sure, that animals didn´t use trees in this area at the survey period, at least not larger groups, as we put mist nets several times in the area of the roost trees of 2007, we also caught Bechstein´s bats there, but they used the trees further north.

**L. 295:** I suggest to start the sentence with something like: “However, despite the turbines being operated for more than 15 years, ...”.

Done

**L. 297:** Replace “possible” with “likely” or “probable”.

Done

**L. 299:** Add commas after “PL” and “turbines”.

Done

**L. 301 – 302:** Consider to change to “The size of the roosting areas was different in both study areas and years, varying between 19 and 105 ha.”.

Done

**L. 304 -305:** “since we didn´t find changes of roosts between all groups of roost trees” – Unclear: do you mean that not all tagged bats always shared the same roosts?

We tried to clarify that, they didn´t always share roosts that´s more or less clear, but furthermore some individuals roosted exlusivly in a group of trees which were never used by the others (lines 456 to 457).

**L. 307:** Replace “those” with “which”.

Done

**L. 312:** Further clarification would be beneficial for the general reader: how does juvenile age influence the duration of consecutive days a roost is occupied?

We added that in the manuscript (lines 467, 468)

**L. 313:** “simultaneously” – do you mean during the emergence counts on one evening?

Yes, we changed the phrase to make it easier to understand.

**L. 315:** Please provide a reference for the statement that 'colony sizes of Bechstein’s bats are documented between 10 and 50.' To my knowledge, colony size typically refers to adults only, so a total of over 80 individuals including juveniles may not be unusual. Nevertheless, I agree that this is toward the upper end of the typical colony size for Bechstein’s bats.

We added the reference and added the fact that rarely higher numbers are documented, too (lines 472-473).

**L. 317 – 318:** Since it is not known whether the colony size fluctuated in between these two years, I would rather write something like “Although the colony changed its roosting area over the 15 years during which the wind turbines were in operation, colony size was similar in the two surveyed years.”.

We agree and corrected that.

**L. 319:**  “… there were 17 bats counted, of which only one individual was tracked. We presume that these 17 bats were only a subgroup of the entire colony.”

Done

**L. 324:** “patterns” instead of “pattern”

Done

**L. 327:** Do the “foraging search areas” refer to the individual foraging search areas here?

Yes, we added “individual”.

**L. 328:** Consider changing “small” to “relatively small”.

Done

**L. 330:** Replace “at a small space” with “in a small area”.

Done

**L. 335 – 336:** Perhaps more precise: “One individual was recorded foraging at a distance of 4 km to its day roost, …”.

Done

**L. 340 – 341:** I suggest a more concise version: “Furthermore, in areas closer to the turbines, bats showed a preference for habitats characterized by larger tree DBH and reduced herb and shrub layer coverage.”

Done

**L. 347 – 349:** Unclear, please rephrase.

We rephrased this, should be clearer now (523-531).

**L. 356 – 358:** I suggest to rephrase and/or split this sentence into two sentences and try to avoid using “difference” and “different” too often.

Done

**L. 358:** Referring to an earlier comment: it would be useful for the reader if you provided details on the “curtailment regimes” of both wind parks, so it is possible to assess why the one is stricter than the other.

We clarified that in the discussion, as well as in the chapter study area (lines 141-148, 551-559).

**L. 360 – 361:** delete “a” before “positive” and “the before “habitats”.

Done

**L. 376:** Despite the mild effects for Bechstein’s bats, I think it would be important to note here that wind turbines in forests may exert more pronounced negative impacts on other bat species, for example noctules, which are known to roost in forests but generally forage at higher altitudes. I appreciate that you highlight in lines 391 – 393 that the extent to which your results can be generalized to other bat species is limited, and that further research is needed.

We added a passage on that in the manuscript, also in reaction to a second reviewer.

**L. 408:** “forests” instead of “froest”.

Done

**Figures**

**Figures 2, 3, 5 and 6:** I very much like the maps and hope they will be displayed/printed in higher quality in the published version of the manuscript.

We will do our best.

**Figure 7:** As I have remarked earlier, consider adding numbers or letters to the subplots for easier referencing (depending on journal requirements).

Letters were added.

**Figure 8:** To increase the visibility of the lines, I suggest to display the raw data points smaller and perhaps in fainter colour.

We changed the colour and size of the points.

**Reviewer #3: General Comment**

This is an interesting study that clearly involved a great deal of field effort, and the data and analysis you present have the potential to make a valuable contribution. However, the manuscript would greatly benefit from a thorough language revision, including attention to sentence flow and punctuation, to improve clarity and readability. In addition, the description of the methods should be made clearer and more transparent so that readers can fully understand and assess your approach. I also noticed that the results of the Bayesian models are not fully presented. It would strengthen the manuscript if you included the main results (e.g., estimates, credible intervals, etc.) in the main text, ideally in a table or figure, and provided the full model outputs in the supplementary material.

General comment: We did a language revision of the whole manuscript by an English native speaker.

Line-specific comments
• Lines 34–35: Besides direct collisions, bats may also die due to barotrauma associated with wind turbines.

Point was adopted.

• Lines 48–49: I think the impact of wind turbines on forest specialists is not only (or primarily) linked to noise disturbance, but also to habitat degradation and fragmentation. Moreover, forest specialists are already heavily impacted by habitat loss from many other anthropogenic pressures unrelated to wind parks, and wind parks add to these cumulative effects.

We stressed the idea of fragmentation and edge effects as cause for the avoidance (lines 49-59).

• Line 53 (and throughout): The subject of sentences sometimes changes in a way that is misleading or unclear. For example: “As maternity roosts Bechstein’s bats use nearly exclusively tree roosts (22–25). Like other forest bats it switches the roosts often…” The subject shifts here in a way that disrupts clarity. I recommend reviewing these passages for consistency.

Sentence was corrected

• Line 105: I would avoid stating that the weight of the transmitter is “harmless” for bats. The cited publication is relatively old, and a recent review (https://onlinelibrary.wiley.com/doi/full/10.1111/mam.12369) highlights that there is a lack of studies explicitly testing the effect of tag weight, and that tags in general can affect bat behaviour and body condition. While 5% is indeed a conservative and commonly used threshold, it should not be presented as harmless.

We cancelled the word harmless and described it as the common threshold as proposed.

• Line 110: The description of how fixes were categorized could be clearer. For example, were all night fixes assumed to represent foraging? Lactating females often return to the roost during the night to nurse their offspring, so some night fixes might actually correspond to nursing rather than foraging.

As described, we stopped taking fixes, when the signal was stationary during the last 3 min interval, so we took one fix of each stationary position. In a second step, we excluded stationary positions presumably linked to nursing behavior. We clarified those steps in the text (lines 218-223).

• Line 124: Abbreviations should be defined at first use. In this case, MCP is only explained later in the text.

Explanation was added at first use.

• Lines 129–132: This sentence is unclear. Were the points chosen randomly using an algorithm in R? Or is R mentioned here simply because it was the software used? Since you already specify later that all analyses were conducted in R, I would suggest avoiding mention here unless there is a particular methodological reason.

The software was used to select mapping points. But as this is not really an important point we deleted it here.

• Line 136: Could you clarify “10 m around what point exactly”? It seems like some detail may be missing here.

We clarified the sentence by using the phrase mapping points instead of points

• Lines 147–148: How did you determine whether bats were inside the roosts? Were you always able to locate the roost itself? This would also help clarify the concern raised at line 110.

We clarified this step (lines 218-223).

• Line 148: If this is the dataset after excluding the positions mentioned above, I would suggest referring to it as the “final dataset” rather than the “total dataset.”

We used the suggested phrase.

• Lines 168–172: I am not entirely sure I follow this part. From what I understand, the 10 datasets contained the same fixes paired with different, randomly generated pseudo-fixes. Is that correct? Or did you randomly split the fixes and pseudo-fixes into 10 subsets? I think this section would benefit from a clearer explanation.

• Lines 193–194: I am unclear about why the draws from different models were merged. Could you provide some justification or explanation for this step?

Both questions are linked and need a longer explanation, which we decided also to deliver in the supplementary material S2, as perhaps other people want to have an answer too:

To assess the sensitivity of the results to the randomness in the assignment of the absence points to presence points (pairing of absence with presence points), we created 10 different data sets, each representing a different random assignment of absence points to presence points . We fitted the model to all 10 data sets and show the 10 different results in the effect plots (10 different 95% credible intervals). However, instead of showing 10 different regression lines, we show an average of all 10 regression lines. Similarly, instead of showing 10 values for each parameter estimate in the table, we averaged over the 10 results. To do so, we merged the 4000 draws from the posterior distributions of each of the 10 models resulting in 40000 values from which we reported the mean and the standard deviation as an estimate and a standard error of the parameter. By doing so, the mean corresponds to the average of the 10 different means, whereas the standard deviation is larger than the average standard deviation of the 10 posterior distributions because the standard deviation of the merged posterior distributions also includes the variance that is due to the sensitivity towards the random assignment of the absence points.

We think that for measuring the sensitivity towards a data treatment (here pairing of presence with absence points) the data must not be changed except for the treatment (i.e. here the selection of absence points). Therefore, we are convinced that our approach is valid because it honestly shows the variance that is due to random selection of absence points.

In the manuscript, we clarified that all data sets contained the same fixes.

• Lines 210 and 223: The maximum distances between roosts reported seem quite high for Bechstein’s bats. I would suggest making it clearer early on (as you do later in the discussion) that you cannot distinguish between different colonies or stable subgroups within colonies.

That´s a good point. We explain now in the sections study area (lines 123-126) and field methods (182-184), that colonies cannto be distinguished. We corrected in the whole manuscript the use of the word colony to all tracked bats and colony sizes to individual numbers. Furthermore we corrected the passage in the discussion where the colony sizes are discussed (lines 468-474).

• Line 315 (and elsewhere): Please be cautious when stating that colony sizes were so high (and similar colony-specific statements later). As you note earlier in the discussion, you cannot conclusively determine whether these were single colonies.

We clarified that, see point before.

• Lines 363–364: You may want to add some discussion on the site fidelity of Bechstein’s bats. Your results suggest the colonies remain in the area, but Bechstein’s bats are known for strong fidelity to roosting areas and may continue using them due to their relative lack of flexibility. Other forest specialists might respond differently, and this contrast could be worth noting.

We added this point in discussion (lines 540-545).

6. PLOS authors have the option to publish the peer review history of their article ([what does this mean?](https://track.editorialmanager.com/CL0/https:%2F%2Fjournals.plos.org%2Fplosone%2Fs%2Feditorial-and-peer-review-process%23loc-peer-review-history/1/010f01994b45485c-33f78457-c4b3-4f36-9017-d558c2e7df37-000000/Lzuur-rr2JGYvGmsXHcIVeELwaOIFg8rvli19XnS_lU=228)). If published, this will include your full peer review and any attached files.


If you choose “no”, your identity will remain anonymous but your review may still be made public.


**Do you want your identity to be public for this peer review?** For information about this choice, including consent withdrawal, please see our [Privacy Policy](https://track.editorialmanager.com/CL0/https:%2F%2Fwww.plos.org%2Fprivacy-policy/1/010f01994b45485c-33f78457-c4b3-4f36-9017-d558c2e7df37-000000/Wwo-Wo1YU-Bgx2INXv6R6Wy1_hPJWDQHj7ymDqvb7C4=228).

Reviewer #1: No

Reviewer #2: No

Reviewer #3: No

[NOTE: If reviewer comments were submitted as an attachment file, they will be attached to this email and accessible via the submission site. Please log into your account, locate the manuscript record, and check for the action link "View Attachments". If this link does not appear, there are no attachment files.]

While revising your submission, please upload your figure files to the Preflight Analysis and Conversion Engine (PACE) digital diagnostic tool, [https://pacev2.apexcovantage.com/](https://track.editorialmanager.com/CL0/https:%2F%2Fpacev2.apexcovantage.com%2F/1/010f01994b45485c-33f78457-c4b3-4f36-9017-d558c2e7df37-000000/e_Y1w9J898dMbQb918F7rSWQjoQ0kAd5wA0SlFSNTOY=228). PACE helps ensure that figures meet PLOS requirements. To use PACE, you must first register as a user. Registration is free. Then, login and navigate to the UPLOAD tab, where you will find detailed instructions on how to use the tool. If you encounter any issues or have any questions when using PACE, please email PLOS at [figures@plos.org](mailto:figures@plos.org). Please note that Supporting Information files do not need this step.
