# Supplementary material for: Habitat use of Bechstein´s bats (Myotis bechsteinii) within wind parks in forests
Source: PLoS One. 2026 Mar 25;21(3):e0344730. doi: 10.1371/journal.pone.0344730 (PMC13016307; doi:10.1371/journal.pone.0344730)
Supplement: Attachment — Submitted filename: 260119 Response to reviewers.docx [file pone.0344730.s009.docx]

The authors have done a good job revising the manuscript in response to the reviewers’ comments. In particular, the language has improved significantly, resulting in much better readability, flow, and clarity in the revised version. In their responses to the reviewers, I would have appreciated more consistent provision of line numbers referring to the revised manuscript, as this would have made it easier to cross-check the implemented changes.

We are sorry for these inconveniences and hope this time everything is correct.

I have a few additional minor comments and questions; otherwise, I believe the manuscript is close to being ready for publication. The line numbers provided below refer to the clean manuscript without tracked changes.

**Responding to the authors’ replies to two of my previous comments:**

**Regarding my initial comment on line 103 concerning radio-tracking during the lactation period:** You state that there is no suitable alternative method to identify maternity roosts of Bechstein’s bats other than radio-tracking during lactation. I would partially disagree with this point, as Bechstein’s bats are highly philopatric during summer and often use the same roosts already in spring prior to parturition in June, as well as during the post-lactation period in late summer. Radio-tracking during these periods would therefore likely identify the same roosting areas, while potentially causing less disturbance during the sensitive lactation period. That said, spatial use patterns do differ between pre-lactation, lactation, and post-lactation phases, and given that you obtained the appropriate permissions, the applied approach is acceptable.

Thank you for this assessment.

**Regarding my previous comment on lines 204–205 about how model fit was assessed as being sufficient:** You respond that you prefer to leave it to the reader to judge how much confidence to place in the model. This is fair; however, in that case I suggest explicitly guiding the reader to where such an assessment can be made, for example by providing a clear reference to the relevant supplementary material.

We added a reference to S2 file, where we show the residuals (line 259).

**Abstract**

**L. 16 – 18:** This sentence, particularly the first part, is somewhat repetitive of the sentence in lines 13–15.

That´s a good point, we removed the second part of the first sentence in line 13-14.

**Introduction**

**L. 63 – 64:** You state that narrow-space foraging bat species may be especially sensitive to noise disturbance. While relevant references are provided, I suggest elaborating on this point in the manuscript itself, so that interested readers do not need to consult the cited literature to understand the underlying reasoning.

We added that (line 62-64)

**Methods**

**L. 102:** I suggest to use “roosted” instead of “settled” here, and perhaps also in line 44 and 113.

We corrected that.

**L. 103 – 104:** You may address this elsewhere in the revised manuscript, but I am still unclear about how turbine operation was restricted in practice. To facilitate comparison among wind parks, it would be helpful to provide more detailed information on the exact algorithms used. While I am not familiar with their precise implementation, I assume they depend on factors such as time of day, wind speed, and possibly season; clarifying this would improve transparency and interpretability.

The algorithms are explained more detailed in lines 115-120.

**L. 170 – 171:** I noticed that the categories are all covering 20%, except for the first one (0-10%). Is there a specific reason for this, or how did you choose these categories?

We tried to use only 10% steps in a former study, but as the categorisation is a manual estimation, it´s hard to reproduce the exact results especially in the middle categories. So we decided to use 10% only in the low categories, where it´s easier to differentiate.

**L. 184:** “for a longer time” – how is that defined?

In field it´s quite obvious that there are two different resting behaviours: It occurs quite often that the bat rests for several (approximately up to 10 min) inside the foraging habitat, we presume that´s often the case after it was successful in catching a prey. And sometimes it flies back in direction of the roosting tree and the signal is stationary for a longer time, approximately more than 10 -15 min which presumably means that the bat nurses the young. As it concerned only few points we didn´t use a strict time cut. For better understanding we changed the expression to “approximately more than 10 minutes” (line 185)

**L. 196 – 198:** were the data of these two individuals then removed from all further analysis?

The data was not removed as this only concerned the 50% LoCoH which was not relevant for the further analysis.

**L. 207:** “next” – I suggest to use “closest” or “nearest” to underline the spatial meaning. Also in line 226.

We changed that.

**L. 220:** “selected” – should this instead be “removed” or “excluded”? It reads like leaf cover was kept, while herb cover and proportion of broad-leaved trees were excluded, but the latter two are listed in lines 225 – 226. Therefore I assume leaf cover was excluded? Please clarify.

Thank you for your exact revision, you are right, there was a mistake, we corrected that (Lines 219-224).

**L. 222:** missing space between “the” and “proportion”.

We corrected that.

**Results**

**Table 1:** I am not entirely familiar with the model used here, and I am somewhat unclear about how this table relates to the results described in the text and those shown in Figure 7. Based on Table 1 alone, I would assume that effects are considered “significant” when their credible intervals (CIs) do not include zero.

We tried not to interpret the results in a dichotomous way (significant vs. non-significant) because such a dichotomous way of interpreting statistical results has led to the publication bias that we need to avoid (see. e.g. <https://www.nature.com/articles/d41586-019-00857-9> or <https://www.tandfonline.com/doi/full/10.1080/00031305.2019.1583913> ). Therefore, we describe the results (in the results section) as they are in our sample (i.e. in tense referring to our data, without pretending that these results have general validity) and we give the CIs so that the results later can be used in meta-analysis with a correct weighting to infer effects of more general validity.

Under this interpretation, “distance to WT,” “distance to roost,” and their interaction appear to have a significant negative effect on preference, along with a positive effect of SD of DBH. The latter, however, is not mentioned in the text, where median DBH is mentioned instead. This interpretation aligns well with Figure 7C and, at least in part, with the results given in the text (lines 330 – 333). However, you also state (lines 334 – 335) that shrub and herb layer habitats were avoided close to turbines, yet the corresponding effects in Table 1 all have CIs that include zero. Likewise, in Figure 7, most CIs for these variables appear to include the Preference = 1 line, which (if my understanding is correct) indicates no preference.

Yes, preference = 1 means no preference.

It is possible that I am mistaken and that the relevant reference value for the credible intervals is not zero (0) but one (1). If so, however, the CIs of all variables would exclude 1 except for “SD of DBH” and the interaction “distance to WT:distance to roost,” which does not fully correspond to what is shown in Figure 7.

Apologies if I am misunderstanding the model output, but I would appreciate a clearer explanation of how Table 1 and Figure 7 are related and how the reported effects should be interpreted.

We added an explanation of how the values in Table 1 are related to Fig. 7 (lines 342-346).

**Discussion**

**L. 410 – 411:** Another reviewer suggested immigration as a possible explanation for why group or colony sizes appeared to remain stable despite avoidance behavior or potential negative effects of wind turbines. However, in Bechstein’s bats, immigration into other colonies is known to be extremely rare (see, e.g., Kerth and van Schaik 2012; Kerth 2022, p. 7f.), making this explanation unlikely.

Kerth, G., and J. Van Schaik. 2012. ‘Causes and Consequences of Living in Closed Societies: Lessons from a Long‐term Socio‐genetic Study on Bechstein’s Bats’. Molecular Ecology 21(3):633–46. doi:10.1111/j.1365-294X.2011.05233.x.

Kerth, Gerald. 2022. ‘Long-Term Field Studies in Bat Research: Importance for Basic and Applied Research Questions in Animal Behavior’. Behavioral Ecology and Sociobiology 76(6):75. doi:10.1007/s00265-022-03180-y.

Yes, you are right. As the explanation relates to forest dwelling bats in general, we don´t remove it completely, but we added, that immigration isn´t very likely for Bechstein´s bats (lines 415 -416)

**L. 440:** “mitigate” instead of “weaken”?

We changed that.

**L. 453 – 458:** I appreciate that you acknowledge that other species may respond differently, and that in lines 503–506 you note that the transferability of your results to other species may be limited and that further research is needed. However, I still feel that the discussion could benefit from at least a brief consideration of the potential risks posed by wind turbines in forests for other bat species (particularly those with different foraging strategies) or for other forest-dwelling taxa (e.g. other mammals or birds), even if these are beyond the primary focus of your study.

We added a sentence about possible reactions of other bat species, which completes the picture (lines 461-463). We feel that it´s beyond the scope of this paper to regard other taxa, too.

**Figures**

**Figures 5 and 6:** Despite the different shades of grey, it is somewhat difficult to distinguish the 95% LoCoHs for the two survey periods. I suggest using different hatching orientations to improve clarity.

We changed that.

**Figure 7:** I already mentioned this in my comment on Table 1, but here it appears that the CIs for almost all variables include the Preference = 1 horizontal line. The exceptions seem to be “Distance to roost” and possibly “SD of DBH,” which also appear to be the only variables deemed “significant,” according to my understanding of Table 1. Could you please clarify and make clear how Table 1 and Figure 7 are related?

Regarding statistical significance, see our answer above. We now explain how Table 1 and Figure 7 are related in the legend of Table 1.
